# Probing layered arc crust in the Lesser Antilles using receiver functions

**DOI:** 10.1098/rsos.180764

**Published:** 2018-11-14

**Authors:** David Schlaphorst, Elena Melekhova, J-Michael Kendall, Jon Blundy, Joan L. Latchman

**Affiliations:** 1 School of Earth Sciences, University of Bristol, Wills Memorial Building, Bristol, UK; 2 Seismic Research Centre, The University of the West Indies, St. Augustine, Trinidad and Tobago

**Keywords:** island arc, crustal structure, seismic properties of rocks, receiver function

## Abstract

Oceanic arcs can provide insight into the processes of crustal growth and crustal structure. In this work, changes in crustal thickness and composition along the Lesser Antilles Arc (LAA) are analysed at 10 islands using receiver function (RF) inversions that combine seismological data with v_P_/v_S_ ratios estimated based on crustal lithology. We collected seismic data from various regional networks to ensure station coverage for every major island in the LAA from Saba in the north to Grenada in the south. RFs show the subsurface response of an incoming signal assuming horizontal layering, where phase conversions highlight discontinuities beneath a station. In most regions of the Earth, the Mohorovičić discontinuity (Moho) is seismically stronger than other crustal discontinuities. However, in the LAA we observe an unusually strong along-arc variation in depth of the strongest discontinuity, which is difficult to explain by variations in crustal thickness. Instead, these results suggest that in layered crust, especially where other discontinuities have a stronger seismic contrast than the Moho, H–k stacking results can be easily misinterpreted. To circumvent this problem, an inversion modelling approach is introduced to investigate the crustal structure in more detail by building a one-dimensional velocity–depth profile for each island. Using this method, it is possible to identify any mid-crustal discontinuity in addition to the Moho. Our results show a mid-crustal discontinuity at about 10–25 km depth along the arc, with slightly deeper values in the north (Montserrat to Saba). In general, the depth of the Moho shows the same pattern with values of around 25 km (Grenada) to 35 km in the north. The results suggest differences in magmatic H_2_O content and differentiation history of each island.

## 1. Introduction

### 1.1. Overview

Subduction zones are regions on Earth where new continental crust is thought to have formed; however, even though the origin of continental crust has been studied for a long time, major details, such as the discrepancy in composition between average continental crust and that beneath many island arcs, remain unclear [1,2]. A better understanding of crustal structure provides insight into the link between subduction processes and the formation of continental crust through arc volcanism.

The Mohorovičić discontinuity (Moho), the boundary between the crust and the mantle, marks a sharp change in seismic velocities that is thought to be due to chemical composition and/or rheology changes. In addition to the Moho, a mid-crustal discontinuity (MCD), in some areas referred to as the Conrad discontinuity [3], can be observed in many subduction environments (e.g. [4,5] and references therein). Even though it is normally found to be the dominant crustal discontinuity, the Moho is sometimes weak and difficult to resolve, especially beneath volcanic arcs [2,4].

Ideally an active source along-arc seismic experiment would be carried out to provide a comprehensive investigation of crustal structure, but long offsets and large sources are needed. Furthermore, this option is expensive and long offsets, which are required to guarantee that the Moho will be visible, might not be easy to acquire in many regions such as curved island arcs (e.g. [4,6]). Passive–seismic observations in a well-monitored arc setting provide an alternative approach. Receiver functions (RFs) and related H–k stacking are now a common method for studying crustal structures (e.g. [2,7–15]).

The H–k stacking method makes use of the difference between P- and S-wave velocities to estimate the depth of discontinuities at which strong changes in seismic velocities occur (termed H) and the average v_P_/v_S_ ratio between the receiver and the discontinuity (termed k; [8]). The ratio of the P- and S-wave velocities can be used to better constrain the average material properties that are present in the crust between the surface and the discontinuity [16]. To investigate crustal thickness, H–k stacking is normally used with the assumption that the largest P-to-S conversions occur at the Moho.

**Figure 1.**
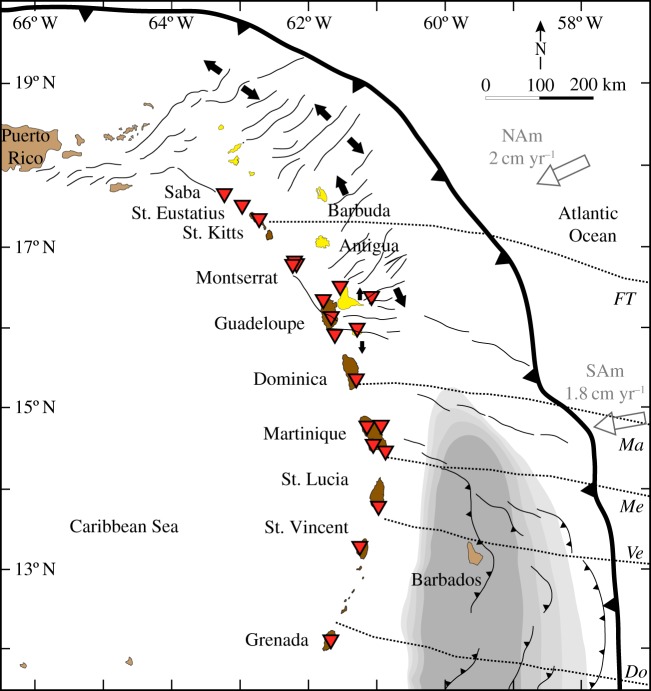
Map of the LAA showing the seismic broadband stations used in this study (red triangles). The western, active branch of the arc is shown in brown; the eastern, inactive branch is shown in yellow. There are 12 stations in Montserrat in close proximity. Plate motion, relative to the Caribbean plate, of the North American (NAm) and South American (SAm) plates is taken from [17]. Sediment thicknesses greater than 10 km are indicated by the grey-shaded area with 2 km contour intervals [18]. Faults indicated by thin black lines [19]; fracture zones by the dotted black lines: FT, Fifteen-Twenty; Ma, Marathon; Me, Mercurius; Ve, Vema; Do, Doldrums [20].

In this work, crustal structure variation along the Lesser Antilles Arc (LAA; figure 1) is studied to investigate potential influences of subduction on the overlying crust. Our approach integrates seismology and petrological observations with a specific emphasis on the LAA. We use extensive seismic data from 26 stations on 10 islands and use RFs to explore crustal discontinuities along the arc. This is complemented by published work on reconstructed crustal structure and compositions of fossil and currently active arcs (e.g. [2,21–23]). The results are compared with structural features and Moho depth estimates from previous works, to propose hypotheses about the link between subduction-related processes and the crustal structure beneath the LAA.

### 1.2. The Lesser Antilles Arc

The LAA (figure 1), extending some 800 km northwards from the South American continent to the Greater Antilles, is an expression of slow (18–20 mm yr^−1^), westward subduction of Atlantic oceanic crust (North and South American Plates) beneath the Caribbean Plate (e.g. [24]). Subduction is sub-orthogonal in the vicinity of Martinique, with sinistral obliquity to the north and dextral to the south [17]. A comprehensive review of the geological and tectonic setting of the LAA is provided by Smith *et al*. [25] and summarized briefly here.

Magmatism along the LAA dates from the Eocene [26]. The present-day arc consists of 11 major volcanic islands; a string of 19 small islands (the Grenadines) lies between St. Vincent and Grenada. Several volcanic centres in the LAA are currently or recently active, including those on Montserrat, Martinique, Dominica and Guadeloupe and the submarine Kick-'em Jenny [27]. The variation in size and spacing of the volcanic islands reflects spatial and temporal variation in magmatic output. Magma production rates in the LAA are at the low end of intra-oceanic arcs worldwide (162 km^3^ km^−1^ Myr^−1^ [28]), possibly a consequence of slow convergence. The LAA lies on the eastern margin of thickened oceanic crust of the Caribbean Plate [29], although the extent to which vestigial Caribbean Plate material, attenuated or otherwise, is present beneath the LAA is not known.

An unusual feature of the LAA is a marked bifurcation north of Martinique into an inactive eastern limb and active western limb; there is evidence for an abandoned, Mesozoic volcanic arc to the west of the Lesser Antilles (the Aves Ridge), separated from the active arc by the Grenada Basin [30]. The westward jump in the northern LAA accounts for a hiatus in volcanism there between mid- and late Miocene times. The cause of the jump is not known. However, there is a marked change in the dip and orientation of the Wadati–Benioff zone along the arc, interpreted by Wadge & Shepherd [24] to indicate that either a single American plate was torn and deformed during subduction or that the North and South American plates were subducted with different velocities. In either case, bifurcation of the LAA just north of Martinique would correspond to the triple junction where Caribbean, North American and South American plates meet.

The division between the northern and southern parts of the LAA is also reflected in the presence and character of the sedimentary cover. The incoming plate in the south is rich in clastic detritus from the South American continent, partially scraped off to form the Barbados accretionary prism (e.g. [31]). To the north, sediment supply is limited by the presence of submarine highs, such as the Tiburon Ridge, and the incoming plate is blanketed by pelagic marine sediments. The spatial variation in sediments plays a role in changing magma chemistry along the LAA [32].

The LAA transects five major fracture zones on the down-going plate (figure 1). The down-going plate displays the topographic expression of strong tectonic extension including normal faults with large amounts of rotation and dome-shaped faulted detachment surfaces, or core complexes, at the edge of the inner valley floor. The presence of serpentine in the down-going plate, associated with fracture zones and/or core complexes, could introduce significant H_2_O to the mantle wedge, perhaps accounting for along-strike variation in magma productivity [33] and subduction zone seismicity [20].

### 1.3. Crustal structure of the Lesser Antilles Arc

The LAA has been the subject of three major geophysical experiments designed to elucidate crustal structure [4,34,35], as well as an attempt to map an along-arc transect of crustal thickness using RFs and H–k stacking [15]. In [12], they have used the same method on the island of Montserrat. Estimates of crustal thickness (depth to Moho) beneath the arc from these studies range from 22 to 37 km. In [4], from their along-arc survey of the southern and central part of the arc (Grenada to Guadeloupe), they reveal the presence of two refractors that split the crust into discrete layers. Their upper layer, with an average velocity of 6.2 km s^−1^, has significant along-strike variation in depth and velocity. The average upper layer thickness is 10 km, but varies from 2 to 20 km [4]. In [36], they interpreted this layer as being built of dense, solidified volcanic rock and plutons of intermediate composition. The uppermost portion of the upper layer has significantly lower seismic velocities and densities, and is likely to be composed of volcaniclastic and sedimentary rocks with abundant fractures [35]. Gravity data from Guadeloupe [37] show that this uppermost layer (v_P_ < 6 km s^−1^) is approximately 4 km thick. The lower crustal layer of [4] that immediately overlies the mantle has average v_P_ = 6.9 km s^−1^ and is thought to represent dense, more mafic igneous rocks, including cumulates.

Two cross-arc seismic surveys, between Dominica and Guadeloupe [35] and south of Grenada [34], provide a more detailed picture of crustal structure. The layering persists, although the overall vertical velocity gradient is smoother than that suggested by Boynton *et al*. [4]. The crust between Dominica and Guadeloupe is 26 km thick and south of Grenada it is 24 km thick. The Moho is not well resolved in either location; mantle v_P_ varies from 7.7 km s^−1^ in the south to 8 km s^−1^ in the centre. Neither survey shows any significant deepening of the Moho beneath the active arc. West of Grenada, the Moho shallows beneath the Grenada Basin (to less than 20 km in places), thickening to 27 km beneath the Aves Ridge. Seismic surveys have been unable to identify unequivocally any vestiges of Caribbean Plate in the sub-arc crust.

Here, we refine the along-arc crustal image of [4] using seismic data collected from 29 remote seismic stations along the active LAA, in combination with insights from experimental and igneous petrology, to develop a method for constraining crustal structure using RF. Our approach, which refines a recent investigation of Moho depths along the LAA using conventional RF analysis [15], has widespread applicability to volcanic arcs and layered crust more generally.

## 2. Receiver functions and H–k stacking

We use seismic broadband stations from different networks, located on most of the major islands of the LAA (figure 1 and table 1); for Montserrat, Guadeloupe and Martinique, more than one station is available. For this study, we limit our catalogue to events greater than magnitude 5.5. Teleseismic events are required (30° to 90° distance) to ensure subvertical incidence angles. Events are filtered using a second-order Butterworth bandpass filter from 0.4 Hz to 3 Hz (after [13]). Only events with a clear P-phase are then selected for this study.

**Table 1.** Information on stations used in this study.

island	station	network^a^	lat (deg)	lon (deg)	no.^b^	time
Saba	SABA	NA	17.6205	−63.2426	11	2008/01/01–2012/30/06
St. Eustatius	SEUS	NA	17.4928	−62.9814	10	2008/01/01–2012/30/06
St. Kitts	SKI	TR	17.3338	−62.7380	2	2008/01/01–2012/10/31
Montserrat^c^	MBBY	MVO	16.6977	−62.2025	3	1999/03/05–2008/01/29
	MBFR	MVO	16.6930	−62.1780	1	2005/06/05–2008/01/29
	MBGB	MVO	16.7323	−62.2278	5	1996/10/19–2008/01/29
	MBGH	MVO	16.7225	−62.2086	9	1996/10/19–2008/01/29
	MBLG	MVO	16.7250	−62.1622	4	2005/03/01–2008/01/29
	MBRY	MVO	16.7039	−62.1532	5	1998/01/30–2008/01/29
	MBWH	MVO	16.7422	−62.1909	1	2005/04/02–2008/01/29
	MBBE	MVO	16.7435	−62.1601	0	1996/10/19–1998/04/12
	MBGA	MVO	16.7102	−62.1886	0	1996/10/19–1998/04/12
	MBGE	MVO	16.6900	−62.1937	0	1996/10/19–1998/01/23
	MBHA	MVO	16.7398	−62.1713	0	2004/09/06–2008/01/29
	MBLY	MVO	16.7171	−62.1841	0	2002/10/31–2006/06/21
Guadeloupe^d^	DHS	WI	16.2887	−61.7652	6	2012/09/26–2013/11/13
	ABD	WI	16.4744	−61.4881	0	2012/09/26–2013/11/13
	CBE	WI	16.0671	−61.6112	0	2012/09/26–2013/11/13
	DSD	WI	16.3128	−61.0661	0	2012/09/26–2013/11/13
	MAGL	WI	15.9494	−61.2822	0	2012/09/26–2013/11/13
	TDBA	WI	15.8550	−61.6354	0	2012/10/29–2013/11/13
Dominica	DLPL	TR	15.3324	−61.2468	15	2008/06/01–2012/10/31
Martinique^d^	FDF	G	14.7350	−61.1463	11	1998/11/25–2012/30/06
	BIM	WI	14.5181	−61.0670	0	2012/09/26–2013/11/13
	ILAM	WI	14.7745	−60.8753	0	2012/12/07–2013/11/13
	MPOM	WI	14.4447	−60.8588	0	2012/11/22–2013/11/13
St. Lucia	MCLT	TR	13.7115	−60.9426	14	2008/01/01–2012/10/31
St. Vincent	SVB	TR	13.2745	−61.2504	8	2008/01/01–2012/10/31
Grenada	GRGR	CU	12.1324	−61.6540	20	2006/12/12–2012/30/06

^a^The networks are: CU, Caribbean Network; G, Geoscope; MVO, Montserrat Volcano Observatory; NA, Netherlands Antilles Seismic Network; TR, Eastern Caribbean Seismograph Network; WI, West Indies French Seismic Network.

^b^Number of receiver functions.

^c^Multiple stations were used on Montserrat, but not all showed good quality events.

^d^Data of multiple stations were observed on Guadeloupe and Martinique, but in both cases one station showed better quality and was therefore used for the analysis.

The method uses the coda of an arriving signal, which contains mode-converted energy due to the structure beneath the receiver [38,39]. A large velocity contrast at a seismic discontinuity leads to a strong P-to-S-converted phase [8]. The signal at the receiver is a convolution of the initial signal with the subsurface structure. Therefore, assuming horizontal layering, a deconvolution can be carried out to remove the source effects and produce a sequence of pulses representing this structure by isolating the P-to-S conversions [8,13,39,40]. This resulting sequence is called a ‘receiver function’ (RF).

In this study, the extended-time multitaper frequency-domain cross-correlation receiver function (ETMTRF [40]) is used to create the RFs. ETMTRF, based on the work of [41], includes later arriving multiple converted phases and has the advantage of being less sensitive to noise. We use a high-frequency cut-off at 1.5 Hz, and for the purpose of this study three overlapping tapers are sufficient. The radial RFs are then stacked by jackknifing [42] from −10 s to + 30 s relative to the P-peak. This produces a standard variation that can be used as a pointwise uncertainty for the RF. Here, the 2*σ* level is used. The H–k stacking method follows the work of [8–10], which involves applying a bootstrapping algorithm [43] to determine the uncertainties of the model parameters.

Based on theoretical arrival times of converted phases, the method derives values for the depth of the discontinuity (H) and the average P-wave to S-wave (v_P_/v_S_ = k) ratio of the crust between that point and the surface. The amplitudes at the theoretical arrival times are summed as follows:

2.1 $$s(H,\;\kappa )=\overset{N}{\underset{n=1}{\sum }}w_{1}r(t_{1})+w_{2}r(t_{2})-w_{3}r(t_{3}),$$

where *N* is the number of RFs used in the stack, *r_n_*(*t*) is the RF amplitude at time *t*, which is the predicted arrival time for the individual phases (the indices are 1 for Ps, 2 for PpPs and 3 for PsPs/PpSs), and *w*_1_, *w*_2_ and *w*_3_ are the weighting factors with $\sum w_{i}=1$.

The weighting factors are chosen so that phases that are more apparent will be enhanced [9,13,44]. During the bootstrapping process, we compute 300 iterations, while changing the value of v_P_ using random, normally distributed values with 95% of the values in a range of Δv_P_ = ±0.3 around a mean of 6.5 km s^−1^. At the same time, the weighting factors are chosen so that 95% fall in Δ*w* = ±0.05 around the means of 0.6 for *w*_1_ and 0.3 for *w*_2_ (with $w_{1}+w_{2}\leq 1$). The best estimation occurs at the location in H–k space where the three phases are stacked coherently [8] among all possible cases in terms of varying v_P_ and the weighting factors.

As the assumed value of v_P_ can also be a source of error [8,14], the mean of v_P_ is changed in subsequent calculations from 6.5 to 6.3 and 6.7 km s^−1^. These values are maximum deviations of the crustal mean v_P_ as determined by Boynton *et al*. [4]. Excluding the noisy part of data from Dominica, our test shows that the bootstrap error is either similar (Martinique, Montserrat, St. Vincent) to the uncertainty due to the change in v_P_ or smaller (in all other cases). In the case of the v_P_/v_S_ ratio, the bootstrap error is always larger. The larger uncertainty between the bootstrap error estimate and the error arising from a change in the mean v_P_ is used in this study.

**Figure 2.**
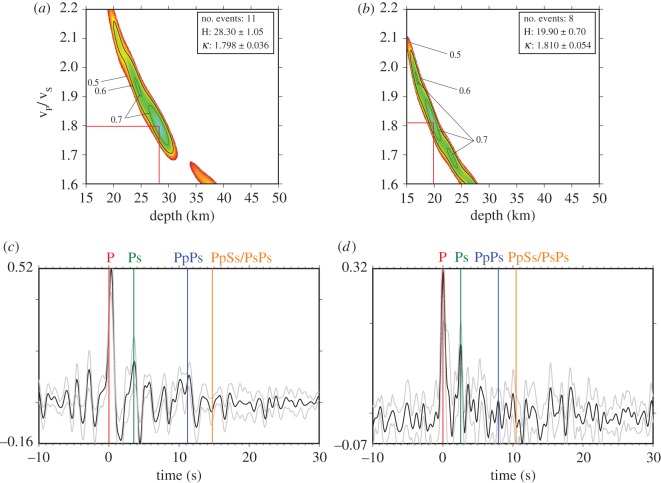
H–k stacking (*a,b*) and RFs (*c,d*) for Martinique (*a,c*) and St. Vincent (*b,d*). In the stacked H–k result, the best fit is indicated by the red lines. The top right values show the number of stacked events, as well as H and k (=v_P_/v_S_) with their uncertainties. The shading shows the normalized amplitude above 0.5. A value of 1.0 means that all iterations result in the same H and k values. In the stacked RFs, the theoretical onset times of the phases, as predicted by the model, are indicated by coloured vertical bars. The grey lines show the pointwise 2σ-jackknife uncertainties. Further H–k stacking results can be found in the electronic supplementary material.

In the case of Martinique, we can see a strong, well-resolved discontinuity at 28.3 ± 1.1 km (figure 2*a*). On St. Lucia, the discontinuity is placed at 46.5 km, much deeper than expected in this area and the solution is very poorly constrained. Furthermore, on some islands H values are arguably too shallow (e.g. St. Vincent with 19.9 ± 0.7 km, figure 2*b*) to be the Moho. We note that the data are in general noisy, as they are from island stations (figure 2*c,d*). To help mitigate this issue, we use the RFs with the highest signal-to-noise (S/N) ratio and employ stacking to further improve the S/N ratio.

An explanation for the unexpectedly shallow or deep results is the existence of a weak Moho beneath some of the islands and a stronger MCD, in which case H–k stacking cannot resolve the Moho, returning instead the depth (H) of the MCD as the preferred result. Additionally, near-surface complexity affects H–k results, often leading to estimates of discontinuities that do not reflect any real structure. We, therefore, conclude that H–k stacking on its own may not be appropriate for mapping layered crustal structure in arc settings without additional constraints. The method is limited by the fact that it will only search for one pair of values. In the case of multiple discontinuities, however, it is likely that peaks in the RF caused by different discontinuities will overlie each other and distort the RF to a point where H–k stacking may find values that do not represent any real discontinuity depth and layer v_P_/v_S_ ratio.

## 3. Inversion for a layered crust

**Figure 3.**
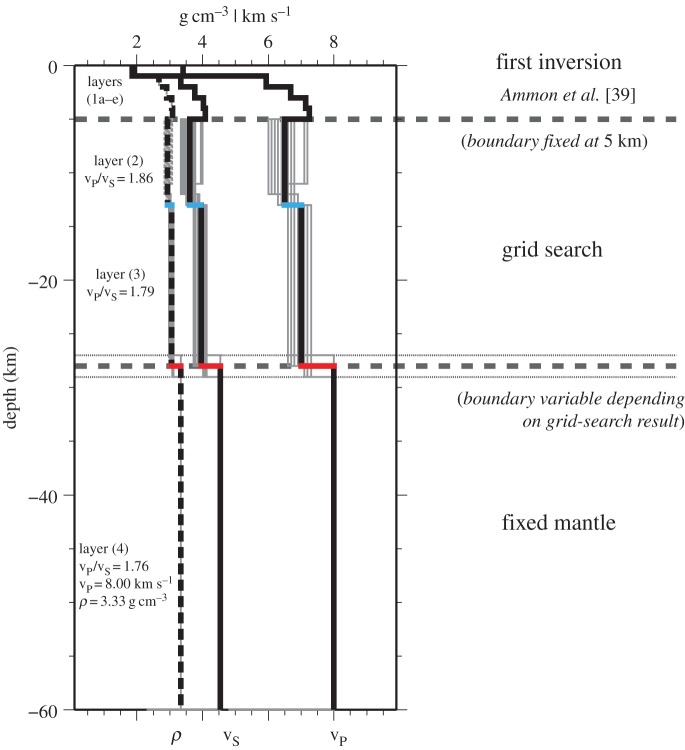
Schematic set-up of the modelling approach. The velocities for the upper 5 km (layer 1a–e, i.e. the shallow crust comprising volcaniclastic rocks and sediments) are modelled using the inversion code of Ammon *et al*. [39]. The following layers (2, 3), i.e. the middle and lower crust, are constrained using the grid search ignoring heuristic demands (see [45]) but including fixed v_P_/v_S_ ratios. The thick vertical lines represent the model with the best goodness-of-fit value, the thinner ones show all models with a goodness-of-fit above 95% to the best model. The thin dotted lines show the possible variability of the Moho in this model. The mantle (layer 4) is kept at fixed values [46]. The example in this figure displays the velocity–depth model for station FDF (Martinique). Note that the decrease in density and velocities below 5 km is a feature of this island and not a result of the inversion code in combination with the grid search. Results from other islands can be found in the electronic supplementary material.

To overcome limitations of the H–k stacking method when applied to layered crust, we adopted a grid-search inversion of a three-layer crust overlying the mantle within the following petrological framework: (i) upper crustal layer composed of loosely consolidated and fractured volcanoclastic sediments and lavas; (ii) middle crustal layer composed of plutonic rocks (solidified magma); (iii) lower crustal layer composed of mafic and ultramafic crustal cumulates; and (iv) mantle layer (figure 3). In a subsequent development of the model, we also consider the presence of vestigial crust of the over-riding, proto-Caribbean plate (layer 2a).

Crustal cumulates are rocks formed by accumulation of near-liquidus phases from magmas undergoing chemical differentiation. Cumulates consist of assemblages that represent instantaneous solid compositions from one or more magma batches. Conversely, plutonic rocks have mineralogy and textures consistent with protracted, *in situ* solidification of magmatic mushes (melt + mineral phases) without attendant differentiation. Our three-layer crustal model is based on studies of currently active and exposed fossil island arcs (e.g. [22,23,34,35,47,48]). The exposed arcs of Talkeetna and Kohistan show lower crust that consists predominantly of mafic/ultramafic cumulates such as pyroxenite, hornblendites and gabbros, whereas the middle crust is composed of evolved, often felsic, plutonic rocks. The lithologies we see in fossil arcs are comparable to those we find in currently active intra-oceanic arcs. Geochemistry and thermobarometry of lavas and their igneous xenoliths along the LAA support that conclusion (e.g. [26,46,49–55]). The seismic studies of active Aleutian and Izu-Bonin island arcs also reveal layered crustal structures which have been similarly interpreted as mafic lower crust and more felsic middle-upper crust based on v_P_ and v_S_ properties of different rock types. We build our model and estimate of v_P_/v_S_ ratios on these observations. The physical properties of the mantle (layer 4) are fixed at: v_P_ = 8.00 km s^−1^, v_S_ = 4.53 km s^−1^, density = 3.33 g cm^−3^. They are derived from [46] and represent putative mantle values beneath the LAA.

Multiple studies of currently active and fossil arcs demonstrated that, although v_P_ and v_S_ vary considerably for the lower-crustal layer, the v_P_/v_S_ ratio is surprisingly constant and on average lies between 1.75 and 1.80 (e.g. [2,21,23,56,57]). A v_P_/v_S_ ratio of 1.79 for the lower-crustal layer (3) is used in our model. The middle crust (2) in our model consists of plutonic rocks of basaltic to andesite compositions. This assumption is based on average lava composition of LAA and upper crustal xenoliths (e.g. [26,50–53]). According to [58], plutonic rocks of this composition will have a v_P_/v_S_ ratio of 1.82 to 1.87 at mid-crustal pressures. The v_P_/v_S_ ratios of the upper layer (1) are controlled primarily by the fracture density and degree of compaction, rather than lithology. In a refinement of our model, we also consider the presence of vestigial Caribbean Plate (layer 2a). The seismic properties of layer (2a) are estimated from rock compositions of the Caribbean oceanic plateau [59]. The v_P_/v_S_ ratio found is 1.86.

The advantage of a fixed v_P_/v_S_ ratio is a significant reduction in parameter space (see the electronic supplementary material for a comparison between modelling with and without petrological constraints). More importantly, v_P_/v_S_ ratios obtained for crustal and mantle lithology allow us to reconcile the seismological and petrological interpretation of crustal structure. To test the reliability of the chosen v_P_/v_S_ ratios, we tested the upper and lower limits of this value for layers (2), (2a) and (3) and found that a modification in the v_P_/v_S_ ratio of ±0.05 does not change the overall discontinuity depth results significantly.

We assume that all melt has either been extracted or is isolated at a very low melt fraction along grain boundaries. Xenoliths from the LAA contain variable, but small, amounts of melt distributed along grain boundaries [49,50,53,55]. We discuss the seismological implication of the presence of melt below.

We set the thickness of layer (1) to be 5 km, consistent with the geophysical data of [34,37,51], and derive its physical properties from the RF data alone. Using the method of [39], we first invert the seismic RF (using −5 s to +15 s after the initial P-peak) for the uppermost 5 km with an initial model that consists of five 1 km thick subsidiary layers (1a–e) and two main crustal layers. We use a smoothness value of 0.1 based on visual observation to create the smoothest models that still fit the observations well. The horizontal slowness was chosen to be 0.06 s km^−1^ (but different values have been tested for stability of the result) and the singular-value decomposition truncation fraction was chosen to be 0.001 to handle values close to zero. The thickness of each layer stays fixed during this inversion. Thicknesses of the middle (2) and lower (3) crustal layers are varied within the range of plausible values throughout different inversion runs to ensure the stability of the solution for layers (1a–e). This first step accounts for the strong effects of the highly variable structure near the surface on the RFs and can overcome the nonlinearity and non-uniqueness of this problem (e.g. [45]). Because of the nature of layer (1) this inversion does not include any petrological constraints. In the second step, we introduce a grid search to investigate the depth to the MCD and the Moho and the velocity contrast at these discontinuities, thus defining the thickness of the middle and lower crustal layers. Having already fixed the highly variable upper layer (1) in a previous step, it is possible to reduce the grid search to a reasonable number of models and computation time. In this step, we introduce v_P_/v_S_ ratios based on the petrological considerations above. We keep the v_P_/v_S_ ratios for individual layers fixed but allow v_P_ and v_S_ to vary. This proves to be a useful constraint, further restricting the explored parameter space. This additional step is needed to vary the thicknesses of the layers, whereas the first step only works with fixed layer thicknesses.

A *χ*^2^-misfit is used to evaluate the match of different models with the seismological data and, thus, make them comparable. It is described by

3.1 $$\chi^{2}=\frac{1}{\sum w(n)}\overset{N}{\underset{n=1}{\sum }}w(n)\times {\left[\frac{d(n)-s(n)}{\sigma (n)}\right]}^{2},$$

where *N* is the number of data points and *d*(*n*), *σ*(*n*) and *s*(*n*) are, respectively, the data RF, its pointwise uncertainties obtained by the jackknife stacking, the model RF at point *n* and a weighting factor *w*(*n*), which is chosen so that it forms an envelope around the maximum at 0 s (P-arrival) and decreases exponentially to both sides (see [45] for further information). The smallest value of *χ*^2^ depicts the best model; the uncertainties on this model are given by all models that reach 95% of this value, taken from a *χ*^2^ distribution table.

We carry out the grid search with v_P_/v_S_ values as high as 2.2 to explore the possible presence of melt, which is known to have a much greater effect on v_S_ than on v_P_ [60]. No low *χ*^2^ solutions are obtained with such large v_P_/v_S_ values, indicating that large pockets of interconnected melt are unlikely beneath the LAA, consistent with the H–k stacking results, as well as [15] and the textural evidence from xenoliths.

## 4. Results

The depth to the MCD and v_P_ for layers (2) and (3) is in excellent agreement with previous work on various segments of the arc [4,12,34,35,61]. The Moho was not observed seismically by Boynton *et al*. [4], but was estimated to lie at about 35 km based on their gravity data. In [15], using conventional H–k stacking, they propose that the average Moho depth beneath the LAA is 29 ± 7 km.

**Figure 4.**
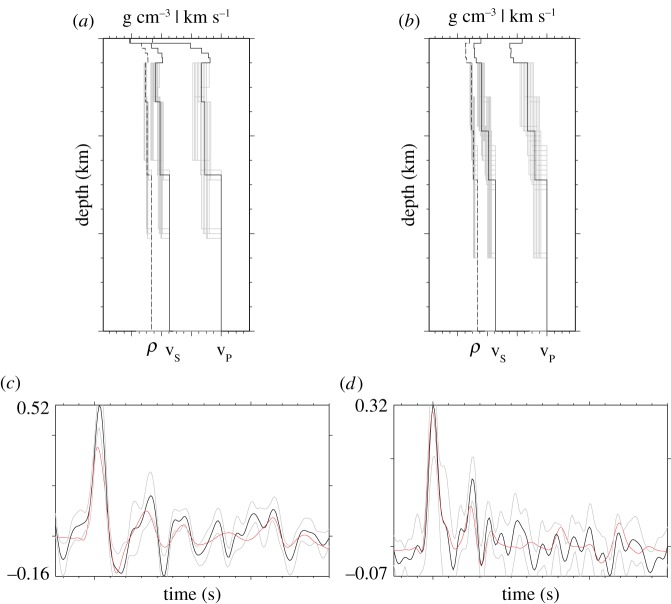
Modelling one-dimensional depth–velocity profiles (*a,b*) and RFs (*c,d*) for Martinique (*a,c*) and St. Vincent (*b,d*). In the profiles, the best model is indicated by the black lines, the grey lines show all models with a goodness-of-fit above 95% to the best model. The RFs show the stacked data RFs (black) and the model RFs (red). The grey lines show the pointwise 2σ-jackknife uncertainties. Further modelling results can be found in the electronic supplementary material.

**Figure 5.**
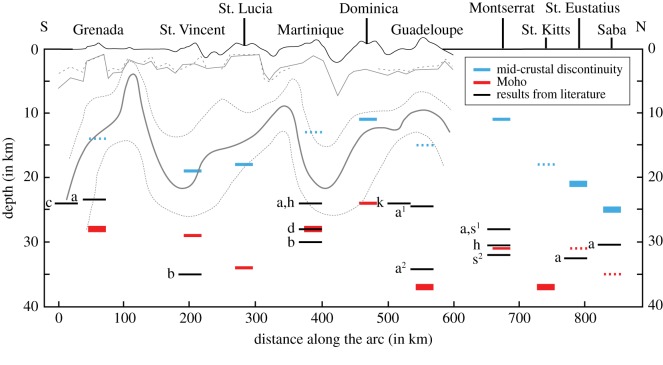
Compilation of inversion results along the LAA from south (Grenada) to north (Saba) showing depths of the MCD and Moho beneath each island based on inversion. The result from the seismic refraction carried out by Boynton *et al*. [4] is shown in the background beneath Grenada to Guadeloupe. The shallower lines represent the upper boundary of the upper main crustal layer and solutions for separate segments of the line (dashed lines); the lower lines show the MCD and its uncertainties (dotted lines). Blue and red horizontal bars denote the best MCD (blue) discontinuity and Moho (red) estimates based on the grid-search inversion. Uncertainties for these values can be accessed in the electronic supplementary material. In the cases of a dominating discontinuity (i.e. a higher S-wave velocity increase than the other) the stronger and weaker discontinuities are indicated by a thick solid bar and a thin dashed bar, respectively. Black horizontal bars indicate Moho depth results from previous studies: a—[15], using a station in the north (1) and one in the south (2) of the island of Guadeloupe; b—[4]; c—[34]; d—[61]; h—J.O.S. Hammond (2011 personal communication); k—[35]; s—[12], showing a distinction using events from the northwest (1) and from the south (2) of Montserrat.

The best fitting models for stations FDF and SVB can be seen in figure 4. The obtained crustal structure (figure 5 and table 2) shows that the depths to the MCD and to the Moho are highly variable over surprisingly short distances of tens of kilometres. In [15], they arrived at a similar conclusion, with up to 10 km change in Moho depth across Guadeloupe alone. Furthermore, we find that the seismic velocities of layers (2) and (3) also vary laterally. Note that uncertainties in the results do not arise from these lateral variations. Although both discontinuities are present along the entire LAA, beneath some islands they compete to produce either a strong Moho and weak MCD or strong MCD and weak Moho (figure 5). For St. Eustatius and Saba, the Moho is very weak but the MCD is very strong. Beneath Grenada, Martinique, Guadeloupe and St. Kitts, the converse is true. The depth to the MCD varies between 11 and 25 km, while the depth to the Moho varies between 24 and 37 km (e.g. Grenada and St. Kitts, respectively).

**Table 2.** H–k and inversion results for each island.

island	no.^a^	H_H–k_ (km)	±_boot_^b^ (km)	±_Δ__v,P_^b^ (km)	k_H–k_	±_boot_^b^	±_Δ__v,P_^b^	v_P,(2)_^c^ (km s^−1^)	*z*_MCD_^c^ (km)	v_P,(3)_^c^ (km s^−1^)	z_Moho_^c^ (km)
Saba	11	23.8	0.4	1.1	1.75	0.02	0.02	6.5	25	7.3	35
St. Eustatius	10	17.1	0.4	0.7	1.73	0.04	0.02	5.8	21	7.0	31
St. Kitts	2	18.2	0.4	0.7	1.72	0.05	0.02	6.3	18	6.7	37
Montserrat	28	33.9	1.1	1.1	1.70	0.17	0.01	7.1	11	7.4	31
Guadeloupe	6	36.4	0.7	2.1	1.80	0.04	0.02	6.4	15	6.6	37
Dominica^d^	7^d(i)^	26.0	0.3	1.5	1.66	0.04	0.02	7.2	11	7.6	24
	8^d(ii)^	14.0	0.8	0.4	1.81	0.10	0.01				
Martinique	11	28.3	1.1	1.1	1.80	0.04	0.01	6.5	13	7.0	28
St. Lucia	14	46.5	1.8	2.1	1.81	0.03	0.01	7.1	18	7.4	34
St. Vincent	8	19.9	0.7	0.7	1.81	0.05	0.03	6.7	19	7.2	29
Grenada	20	25.9	0.7	1.1	1.70	0.04	0.01	6.5	14	6.9	28

^a^Number of receiver functions.

^b^Uncertainty estimations are shown for both the bootstrap error (boot) and the change in v_P_ (Δv,P).

^c^Only values of the best solution are used. See the electronic supplementary material for uncertainties.

^d^Station DLPL shows strong variation with back azimuth. (i) Excluding events from the NW; (ii) only events from the NW—the quality of these was not good enough to perform further investigation.

Our inversion approach considers only new, magmatic arc crust. We have not considered thus far the possible presence of vestigial proto-Caribbean crust (pCc) within the arc (layer 2a). The estimated seismic properties of this layer (v_P_ = 7.11 km s^−1^, v_S_ = 3.97 km s^−1^, v_P_/v_S_ = 1.79) are described above. Incorporating layer (2a) into our models does not change the depths of the discontinuities in the inversion results because the change in seismic velocity between the pCc and the adjacent crust is too small to be identified by RFs and, hence, H–k stacking and crustal inversions. This conclusion comes from models that included an 8 km layer (2a), consistent with the seismic refraction study of [34] beneath the Grenada basin where the pCc is appreciably thinned. In the unlikely scenario that layer (2a) is chosen with sufficient thickness that it takes up most of the crustal column, the MCD is suppressed, leading to a significantly different inverted crustal structure. In our models, a 20 km layer (2a) is found to cause such a change. However, the resultant misfit between the model and data RFs is considerably larger in those instances, leading to the reasonable conclusion that a pCc, if present, cannot exceed thicknesses of around 10–15 km, depending on the island, and may not be present at all.

A comparison of the depth estimates of the crustal discontinuities with those obtained from a range of other studies along the LAA reveals a good match. For example, our estimate of the Moho depth beneath Martinique is around 29 km, which agrees well with independent estimates from [4,61]. The derived MCD depth agrees with estimates from [4] on every island (figure 5). The Moho depth beneath Grenada, Martinique, Dominica and Montserrat agrees with estimates of [4,12,15,34,35,61]. A particularly interesting comparison is with the results of [4], which also show a highly variable crustal structure with an undulating MCD, albeit of greater amplitude (figure 5).

Our preferred final, four-layer velocity model (figure 5) for the LAA is as follows. The 5 km thick upper layer (1) has highly variable v_P_ that we attribute to lithological heterogeneity due to the layering of sediments and volcanics. The values have been used as a correction for the subsequent grid search and the highly heterogeneous nature would need a more detailed investigation in the future to draw further conclusions. P-wave velocities are 5.8–7.2 km s^−1^ in the middle crust (layer 2) in the depth range of 5–25 km, and 6.6–7.6 km s^−1^ in the lower crust (layer 3) in the depth range of 24–37 km. Our RF inversion model, incorporating constraints on v_P_/v_S_ based on crustal lithology, enables us to identify two crustal layers in a way that conventional H–k stacking does not. Because of the changing relative strengths of MCD and Moho, conventional H–k stacking would instead yield only the stronger of the MCD or Moho at each location, giving the illusion of even larger lateral gradients in the depth of a single discontinuity as it switches from MCD to Moho and back again.

## 5. Discussion and conclusion

There are different potential causes for highly varying discontinuity depths and strengths over short distances. In the LAA, we can rule out a changing amount of subducted sediments. The subduction is sediment rich in the south, gradually becoming more sediment poor towards the north, which does not match the pattern observed. The most probable explanation, however, is short-wavelength variability in the delivery of water to the arc, which in turn affects the temperature, composition and volumes of the magma added to the crust. Variations in magmatic water contents of mantle-derived basalts influence their phase relations and consequently the mineralogy and seismic velocities of associated cumulate layers. Similarly, increased addition of water to the mantle wedge beneath the arc will trigger enhanced melt production. Thus, in principle, variations in the delivery of water to the arc can effect changes in both MCD and Moho depths. We tentatively note a spatial correlation with subducting transform faults (figure 1), which are likely to be water rich and serpentinized. The spatial variability in seismicity along the arc has also been attributed to the effects of the subducting fracture zones [20]. The dramatic variations in crustal properties might suggest complicated upper mantle wedge dynamics, which would explain why the seismic properties of the upper mantle wedge beneath the LAA appear to be isotropic [62,63].

The v_P_/v_S_ ratios used in the inversion are based on samples of melt-free material. In contrast to many other areas where RF studies have been carried out, a volcanic arc may be prone to higher melt content in the crust. Partial melt in the crust can lead to higher v_P_/v_S_ ratios to values of up to 2.0 [14]. However, H–k stacking results from stations where the result agrees with results from the inversion and previous methods show values lower than 1.9 on Montserrat and Martinique. The presence of melt in any of the modelled layers will increase the v_P_/v_S_ ratio. The magnitude of this effect will vary with the amount and physical distribution of the melt. Melt-rich layers could be investigated explicitly using our methodology, by assigning a specific, elevated v_P_/v_S_ ratio to a layer. Where melt fractions are very low or melt lenses very thin, these layers will not be readily detectable using RF methods alone. The fact that we see v_P_/v_S_ ratios consistently below 2.0 in the LAA suggests that melt fractions are consistently low. Furthermore, petrological observations show that quartz is a very minor component in all Antilles rocks due to the mafic nature of the arc. Therefore, its contribution to the ratio is negligible and the melt-free model applied in this study seems appropriate for this inversion. This should not be taken to mean that melt is absent beneath the active LAA, simply that where present it occurs in relatively small, disconnected pockets that lie outside the resolution of seismic methods.

We have elucidated along-strike variation in crustal structure in the LAA using an approach that integrates seismology and petrology. The first important outcome of this study is that using a combination of local networks it is possible for the first time to get a detailed study of crustal structure of all major islands in the LAA. Secondly, our approach affords several advantages over a purely seismological approach, especially in arc settings at stations with high amounts of noise, where the H–k stacking method is prone to ambiguity when used without additional constraints. Consequently, the results are supported by data from seismology and petrology and show models that are consistent with each. Based on our results and previous work in other arcs, we conclude that arc crust is highly variable laterally. The strength of the Moho varies along the LAA. It is the dominant discontinuity beneath four islands (St. Kitts, Guadeloupe, Martinique and Grenada), whereas the MCD is dominant beneath two (Saba, St. Eustatius). The MCD can be found at depths between 10 and 25 km (consistent with [4]) while the Moho depth varies between 25 and 45 km, with both discontinuities being located at greater depths in the northern part of the arc. The highly variable nature of both discontinuities can be explained by lateral variation in the mechanisms of melt generation and differentiation along the arc arising from instabilities along the mantle–slab interface. However, more work is needed (e.g. including petrological constraints from further islands) before a more detailed interpretation is possible.

In this study, the advantages of a modelling technique combining seismological with petrological results over a purely seismological approach have been demonstrated. The approach is particularly useful when the crust is lithologically layered. In arc settings at stations with high amounts of noise, the H–k stacking method that was used to investigate the crustal thickness is prone to misinterpretation when used without additional constraints. Models that are derived from a combined grid-search inversion can help interpret results from RFs and H–k stacking.

## Supplementary Material

Compilation of supplementary material

## Supplementary Material

Table of events

## Supplementary Material

Infographic

## Data accessibility

Most data are available in open repositories (http://ds.iris.edu/ds/nodes/dmc/data/types/events/), the data for Montserrat are stored by the British Geological Survey (http://www.bgs.ac.uk). The ETMTRF code (https://github.com/ghfbsd/sacbook/tree/master/methods/rf) and the inversion code (http://eqseis.geosc.psu.edu/~cammon/HTML/RftnDocs/rftn01.html) are publicly available.
