# Supplementary material for: Probing layered arc crust in the Lesser Antilles using receiver functions
Source: R Soc Open Sci. 2018 Nov 14;5(11):180764. doi: 10.1098/rsos.180764 (PMC6281922; doi:10.1098/rsos.180764)
Supplement: Compilation of supplementary material [file rsos180764supp1.pdf]

Supplementary Figures for "Probing layered arc crust in the Lesser Antilles using Receiver Functions"

This document contains:

- a comparison of H–κ stacking sensitivity to different discontinuity strengths,
- H–κ stacking results for all islands,
- 1-D velocity–depth profiles for all islands in combination with a comparison of the model RF to the data RF,
- a comparison of modelling results with and without petrological constraints for station DHS,
- a compilation of modelling uncertainties.

The figures are sorted alphabetically: DHS (Guadeloupe), DLPL (Dominica), FDF (Martinique), GRGR (Grenada), MCLT (St. Lucia), Montserrat (a combination of all stations), SABA (Saba), SEUS (St. Eustatius), SKI (St. Kitts), and SVB (St. Vincent).

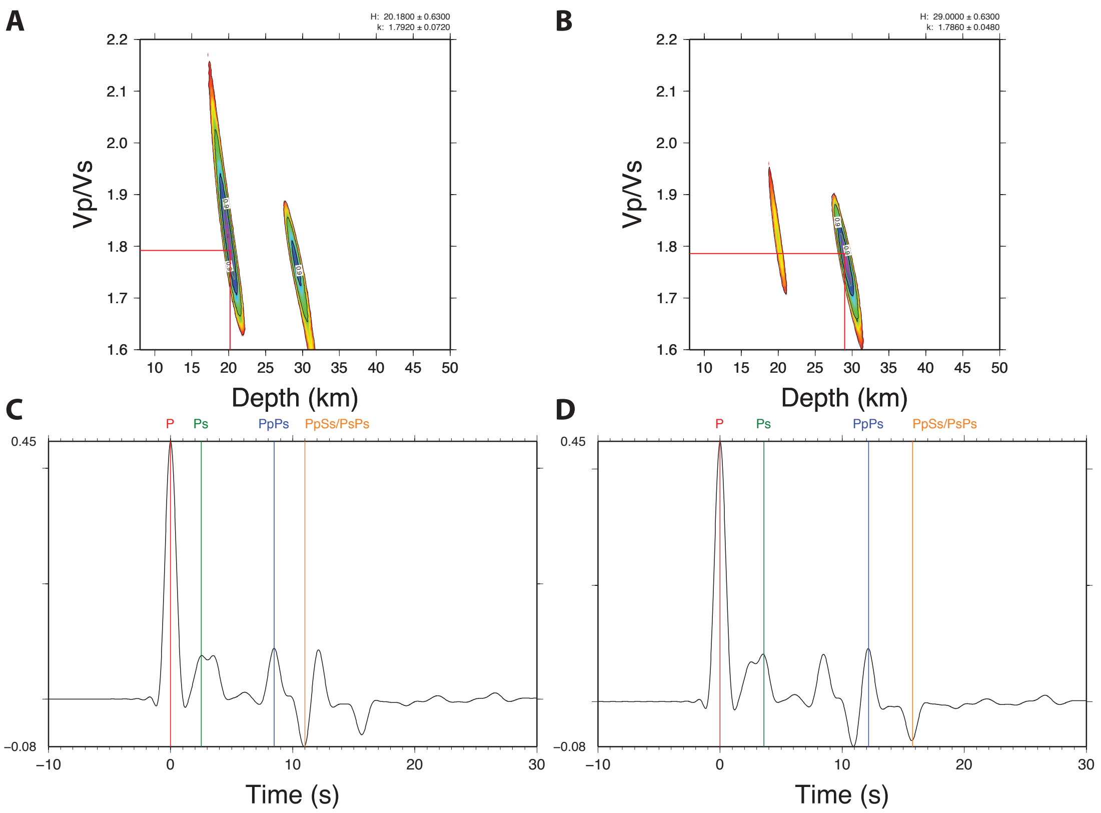

Supplementary Figure 1: H–κ stacking (A, B) and RFs (C, D) of a 3-layer model. The thickness of the first layer is 20 km and the thickness of the second layer is 10 km. Case 1 (A, C): All layers have the same v_P/v_S ratio of 1.73. Other values are: $v_{P,1}$ = 6.2km/s, $v_{P,2}$ = 7.1km/s, $v_{P,3}$ = 8.0km/s. This setup creates two equally large increase in v_P (i.e. two discontinuities of exactly equal seismic strength). It can be observed that the upper discontinuity dominates the result but the second discontinuity can be resolved as well. Case 2 (B, D): Similar to case 1 but here $v_{P,2}$ = 7.05km/s, which creates a slightly stronger deep discontinuity.

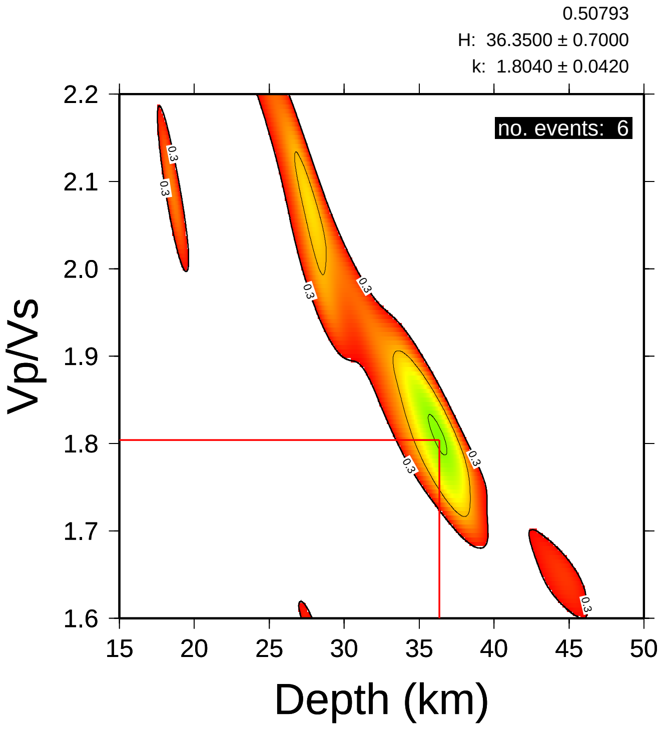

Supplementary Figure 2: H–κ stacking result for DHS (Guadeloupe). The value at the top right of the figure indicates the coherency of all 300 iterations (a value of 1.0 occurs if all iterations show the same best values for H and κ). The best solution is indicated by red lines; the values are also shown in the top right. The number of stacked events is shown in the black box.

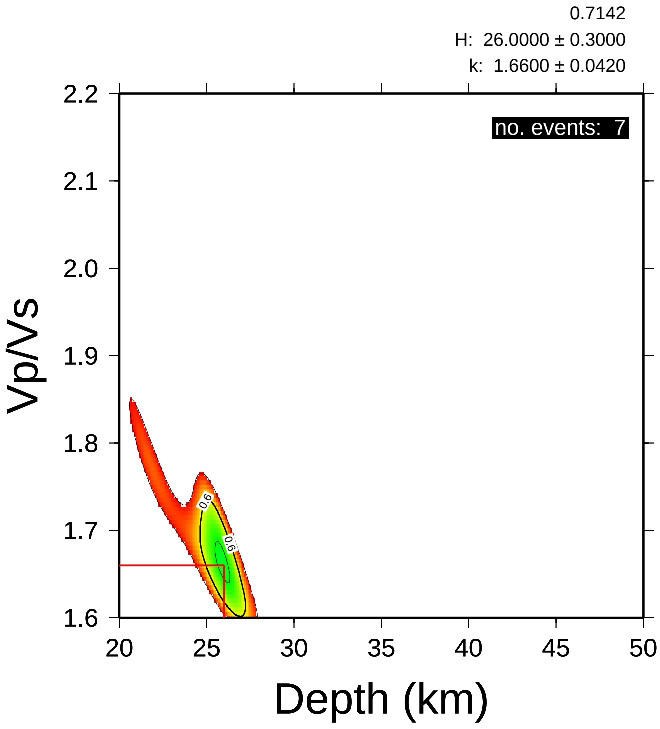

Supplementary Figure 3: H–κ stacking result for DLPL (Dominica), all events except from NW direction. See Supplementary Figure 2 for further information.

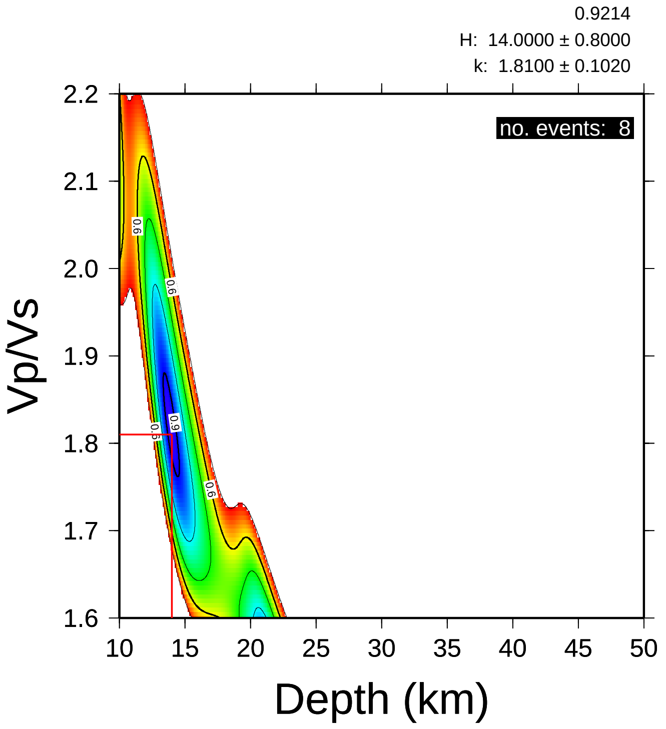

Supplementary Figure 4: H–κ stacking result for DLPL (Dominica), only events from NW direction. See Supplementary Figure 2 for further information.

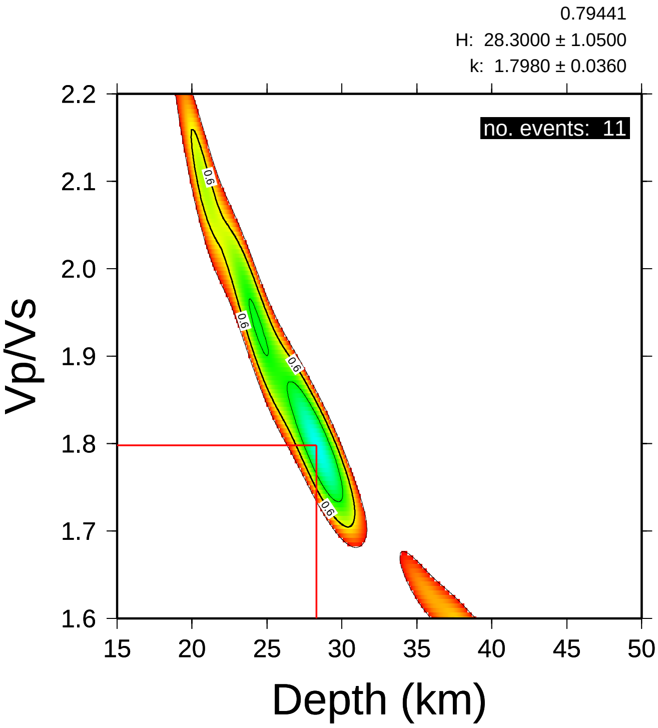

Supplementary Figure 5: H–κ stacking result for FDF (Martinique). See Supplementary Figure 2 for further information.

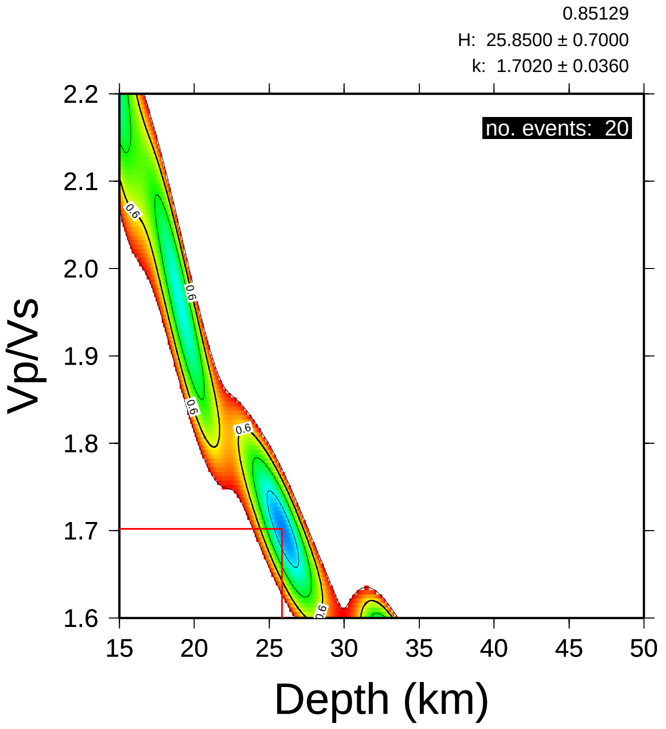

Supplementary Figure 6: H–κ stacking result for GRGR (Grenada). See Supplementary Figure 2 for further information.

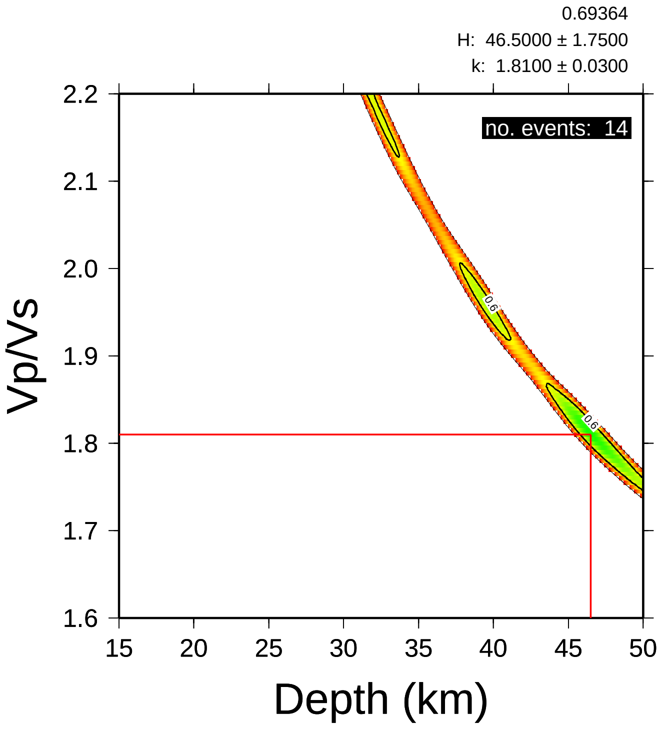

Supplementary Figure 7: H–κ stacking result for MCLT (St. Lucia). See Supplementary Figure 2 for further information.

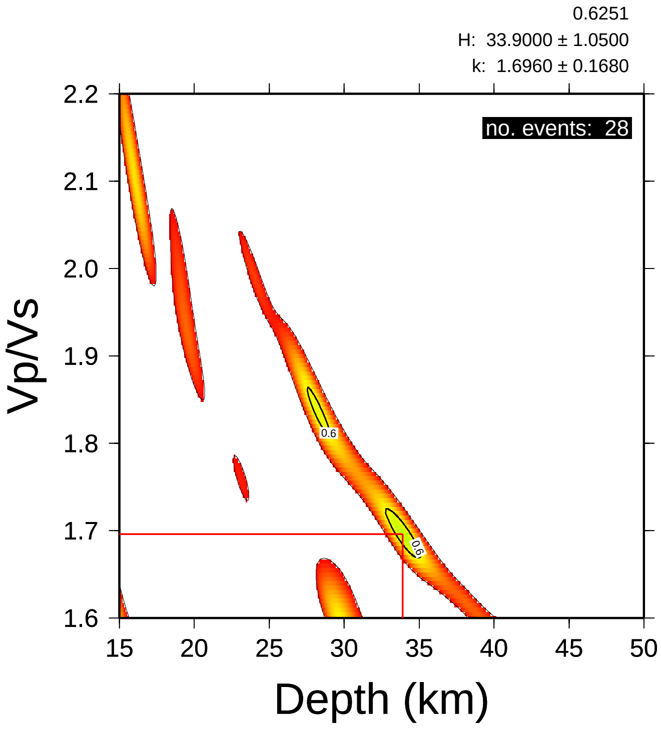

Supplementary Figure 8: H–κ stacking result for all Montserrat stations combined. See Supplementary Figure 2 for further information.

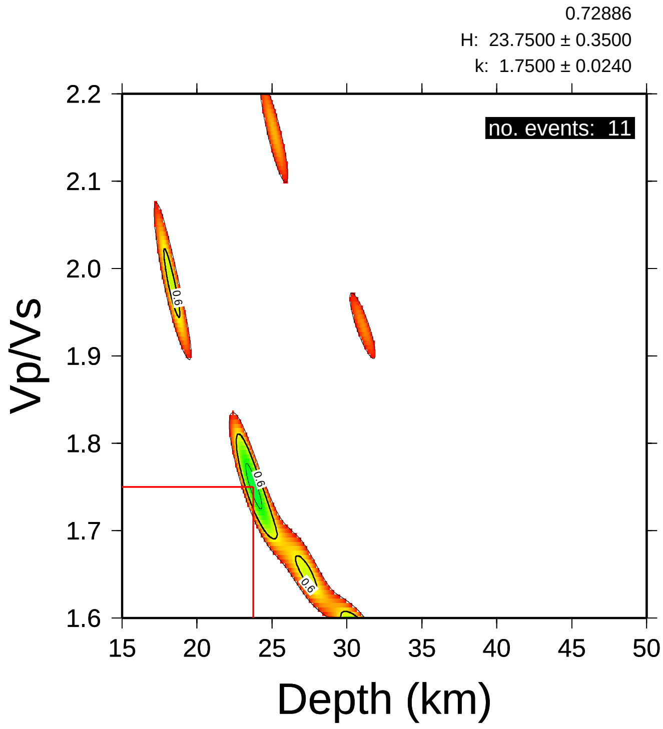

Supplementary Figure 9: H–κ stacking result for SABA (Saba). See Supplementary Figure 2 for further information.

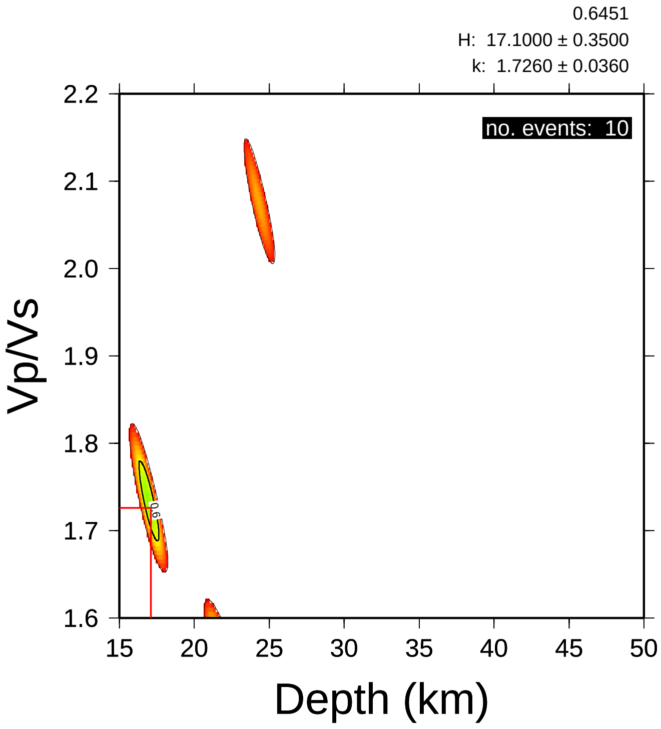

Supplementary Figure 10: H–κ stacking result for SEUS (St. Eustatius). See Supplementary Figure 2 for further information.

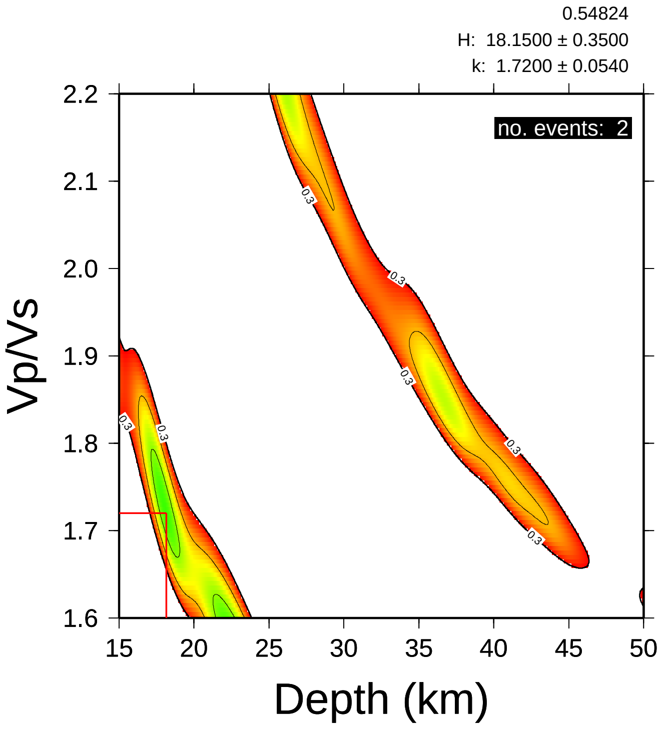

Supplementary Figure 11: H–κ stacking result for SKI (St. Kitts). See Supplementary Figure 2 for further information.

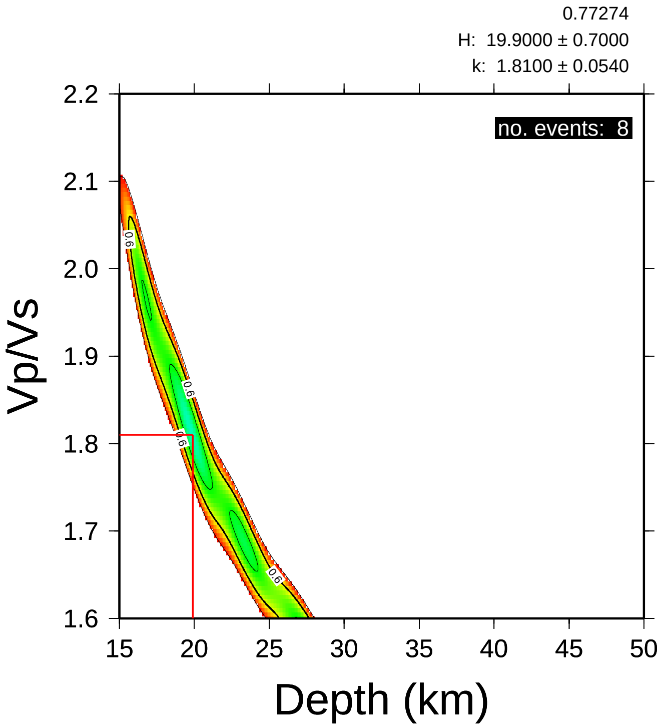

Supplementary Figure 12: H–κ stacking result for SVB (St. Vincent). See Supplementary Figure 2 for further information.

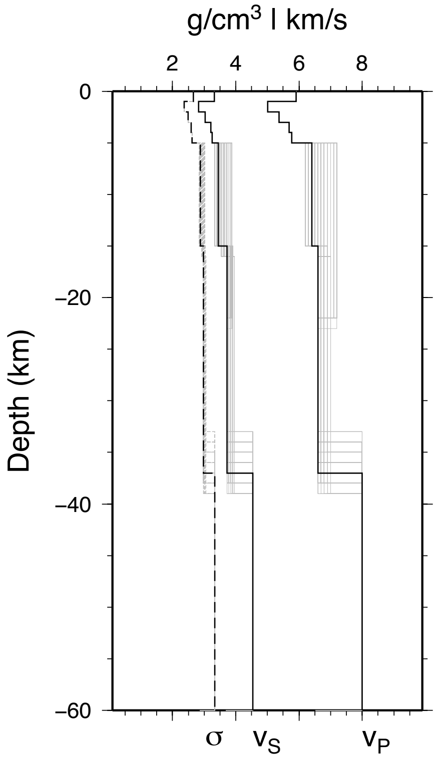

Supplementary Figure 13: 1-D modelling profile for DHS (Guadeloupe). The black lines represent the model with the best goodness-of-fit value, the grey ones show all models with a goodness-of-fit above 95% to the best model.

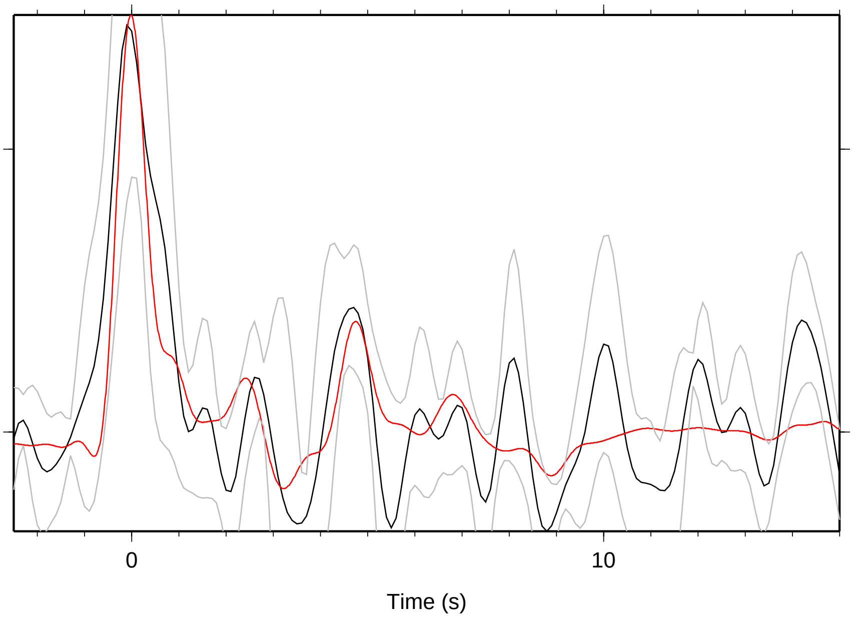

Supplementary Figure 14: Comparison of data RF (black) and model RF (red) for DHS (Guadeloupe). The grey lines show the pointwise 2σ-jackknife uncertainties.

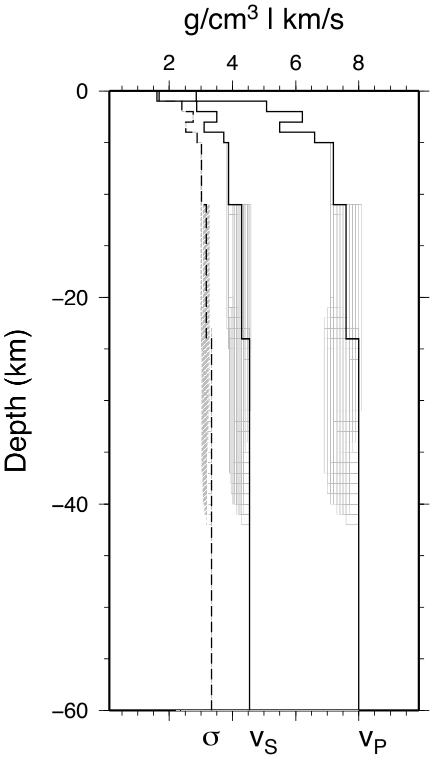

Supplementary Figure 15: 1-D modelling profile for DLPL (Dominica), all events except from NW direction. See Supplementary Figure 13 for further information.

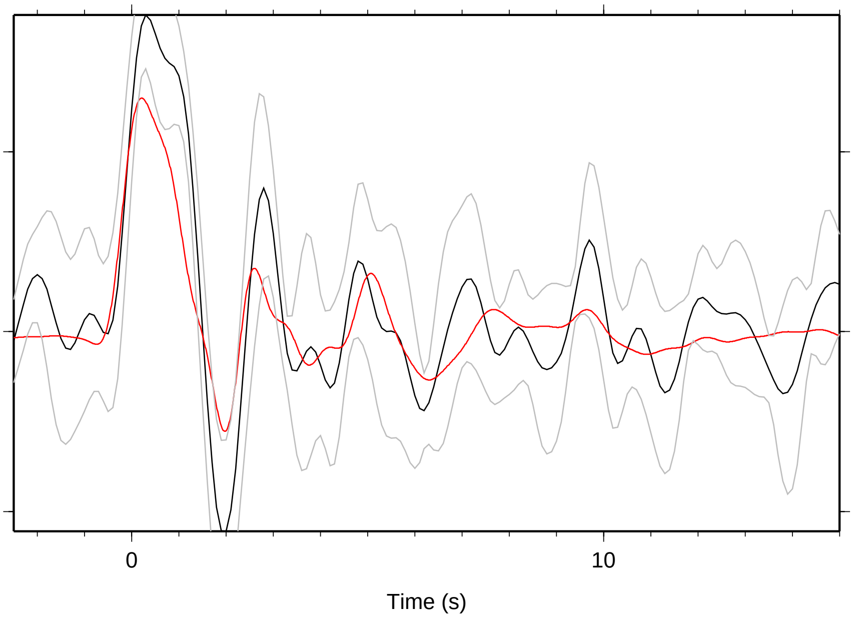

Supplementary Figure 16: Comparison of data RF (black) and model RF (red) for DLPL (Dominica), all events except from NW direction. The grey lines show the pointwise 2σ-jackknife uncertainties.

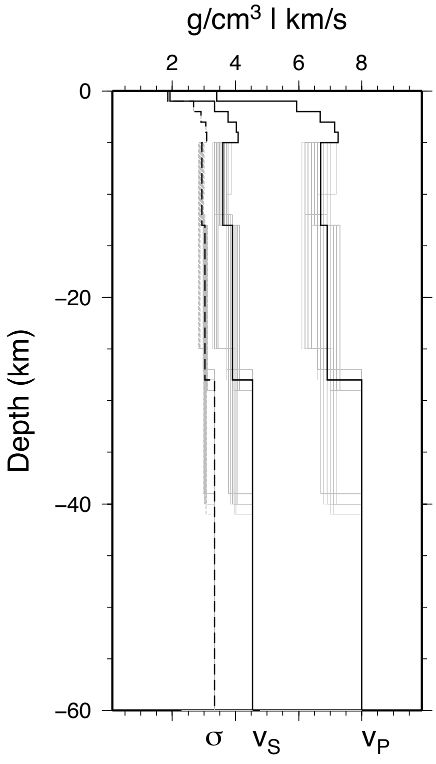

Supplementary Figure 17: 1-D modelling profile for FDF (Martinique). See Supplementary Figure 13 for further information.

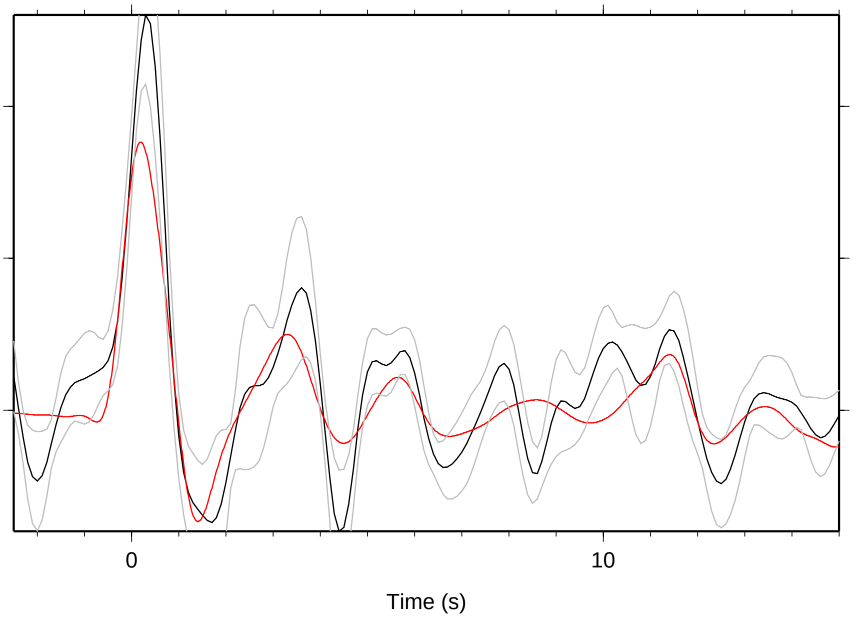

Supplementary Figure 18: Comparison of data RF (black) and model RF (red) for FDF (Martinique). The grey lines show the pointwise 2σ-jackknife uncertainties.

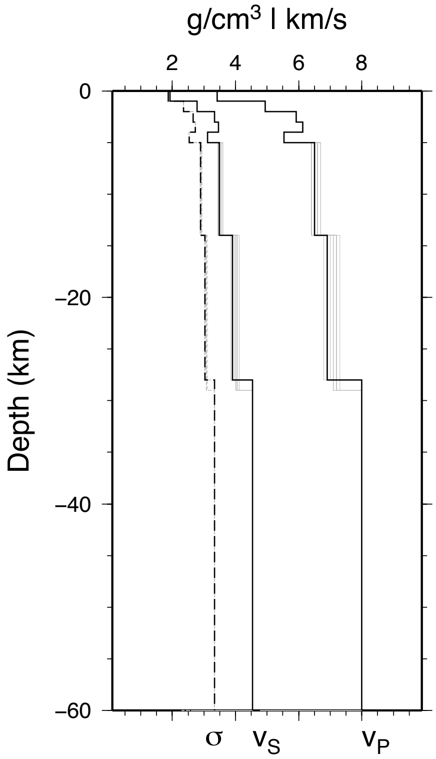

Supplementary Figure 19: 1-D modelling profile for GRGR (Grenada). See Supplementary Figure 13 for further information.

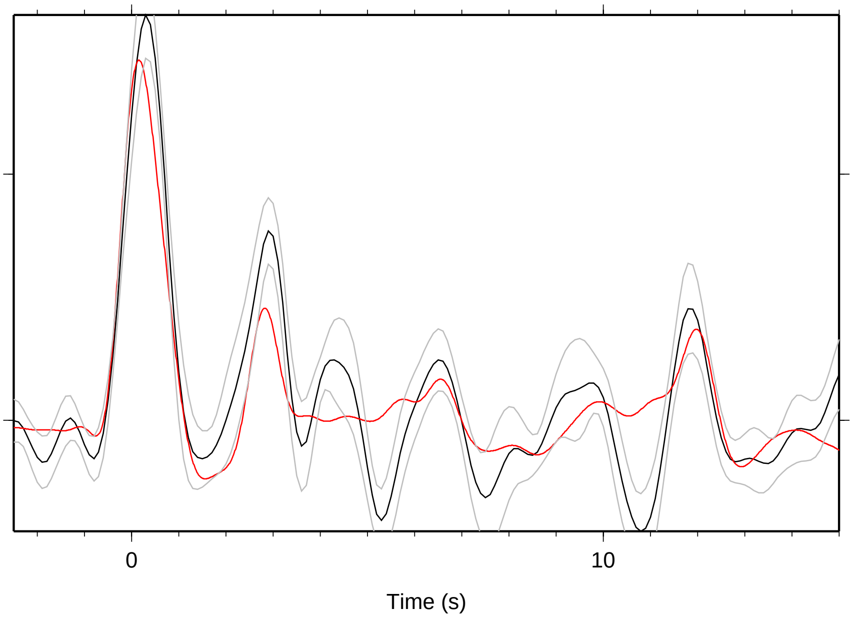

Supplementary Figure 20: Comparison of data RF (black) and model RF (red) for GRGR (Grenada). The grey lines show the pointwise 2σ-jackknife uncertainties.

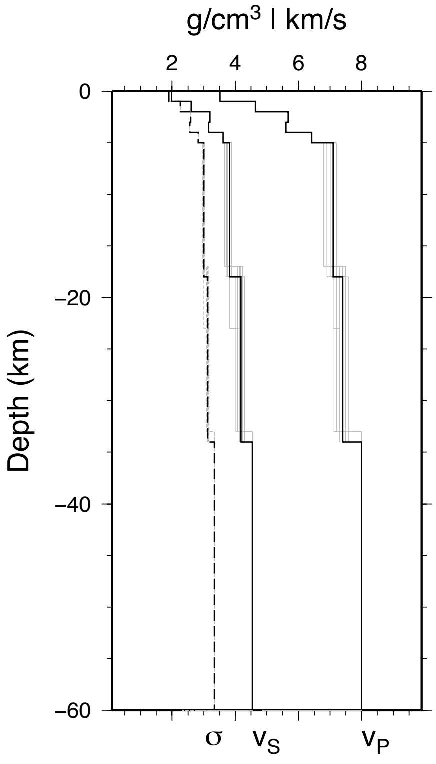

Supplementary Figure 21: 1-D modelling profile for MCLT (St. Lucia). See Supplementary Figure 13 for further information.

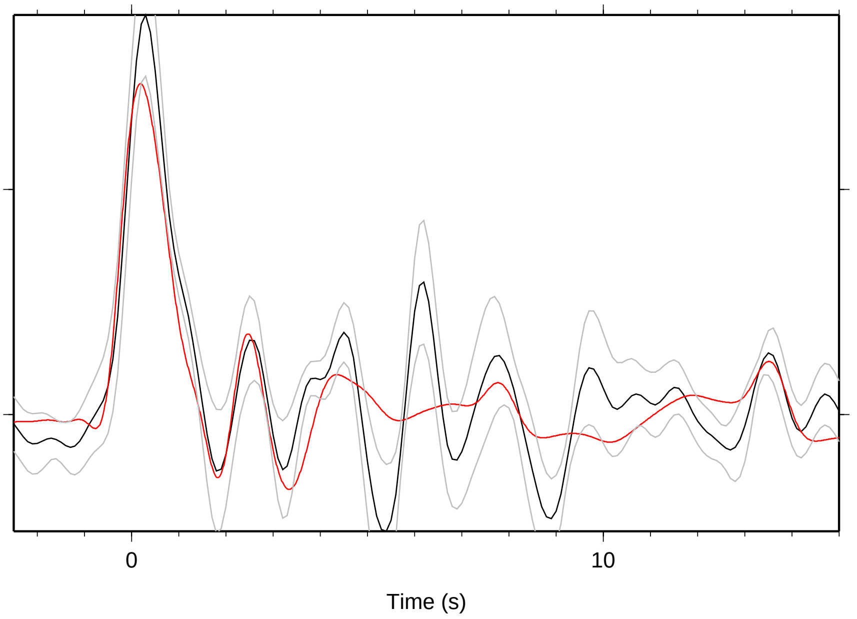

Supplementary Figure 22: Comparison of data RF (black) and model RF (red) for MCLT (St. Lucia). The grey lines show the pointwise 2σ-jackknife uncertainties.

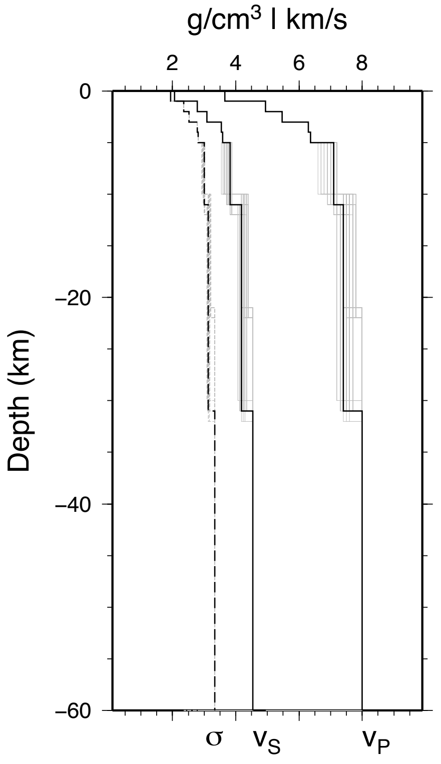

Supplementary Figure 23: 1-D modelling profile for all Montserrat stations combined. See Supplementary Figure 13 for further information.

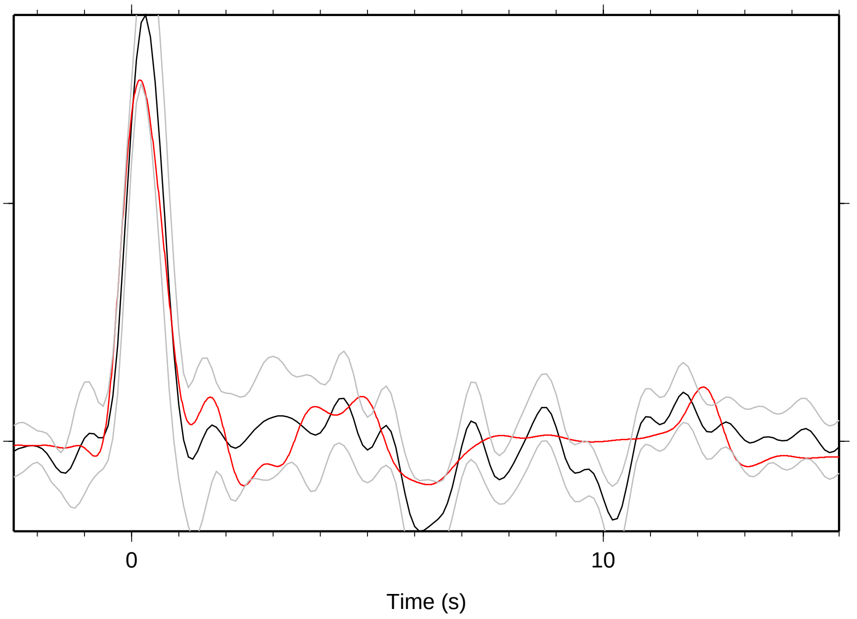

Supplementary Figure 24: Comparison of data RF (black) and model RF (red) for all Montserrat stations combined. The grey lines show the pointwise 2σ-jackknife uncertainties.

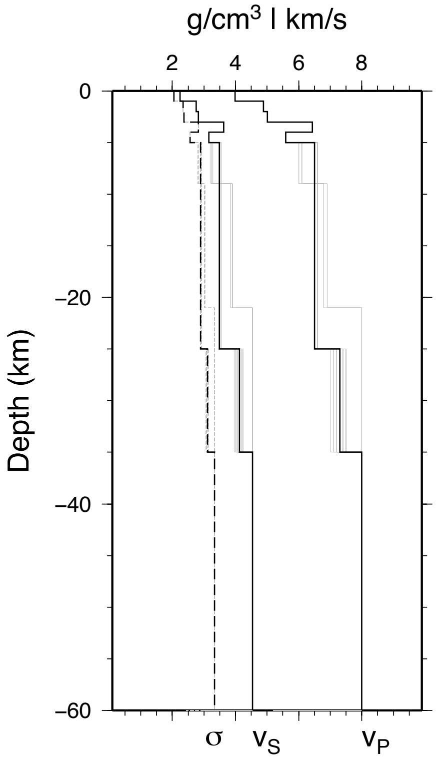

Supplementary Figure 25: 1-D modelling profile for SABA (Saba). See Supplementary Figure 13 for further information.

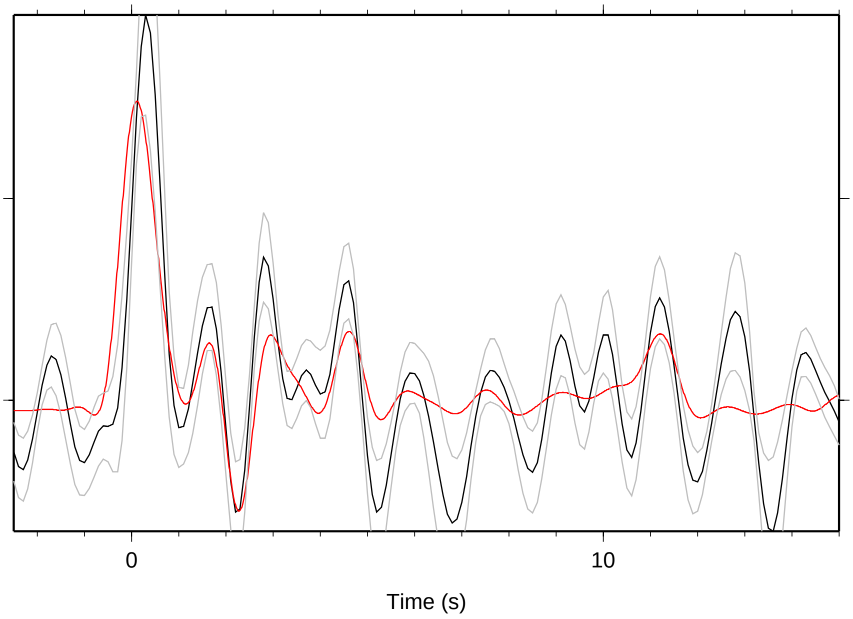

Supplementary Figure 26: Comparison of data RF (black) and model RF (red) for SABA (Saba). The grey lines show the pointwise 2σ-jackknife uncertainties.

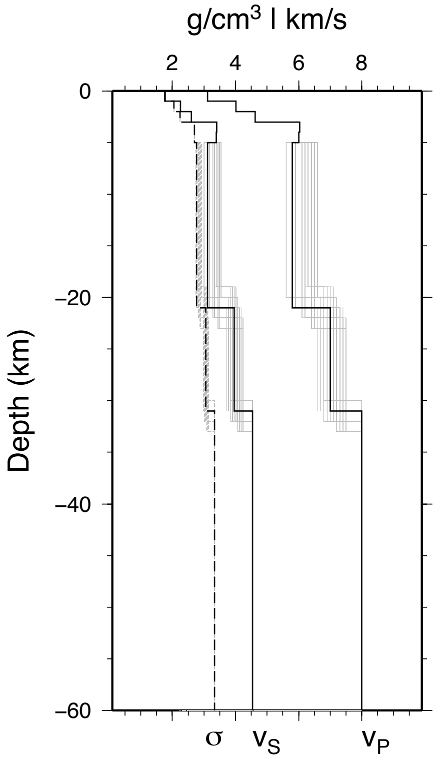

Supplementary Figure 27: 1-D modelling profile for SEUS (St. Eustatius). See Supplementary Figure 13 for further information.

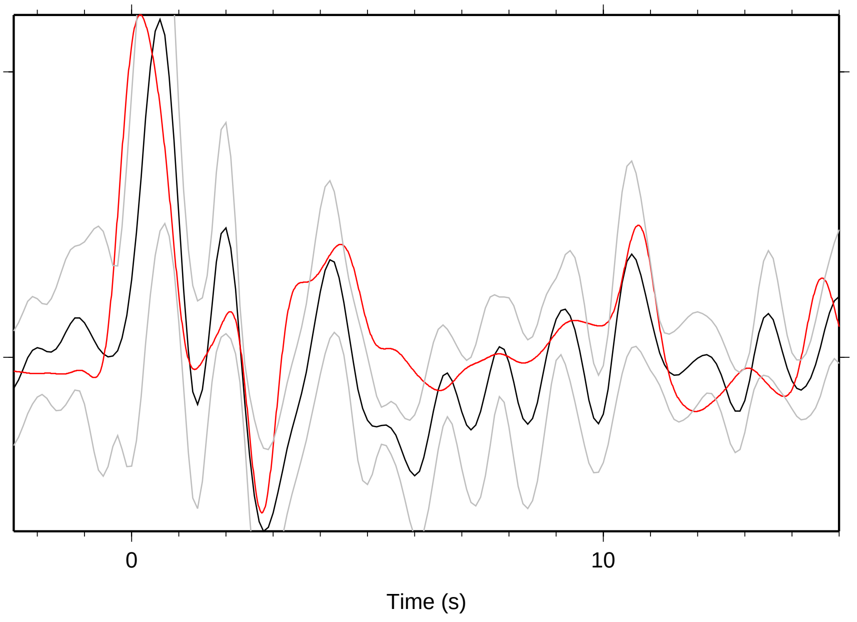

Supplementary Figure 28: Comparison of data RF (black) and model RF (red) for SEUS (St. Eustatius). The grey lines show the pointwise 2σ-jackknife uncertainties.

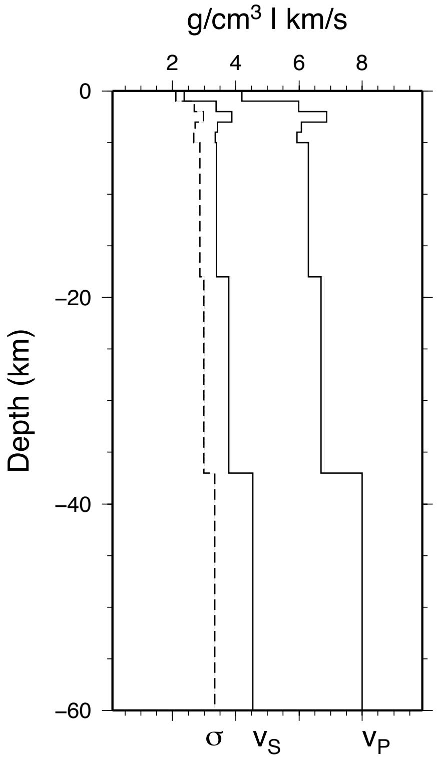

Supplementary Figure 29: 1-D modelling profile for SKI (St. Kitts). See Supplementary Figure 13 for further information.

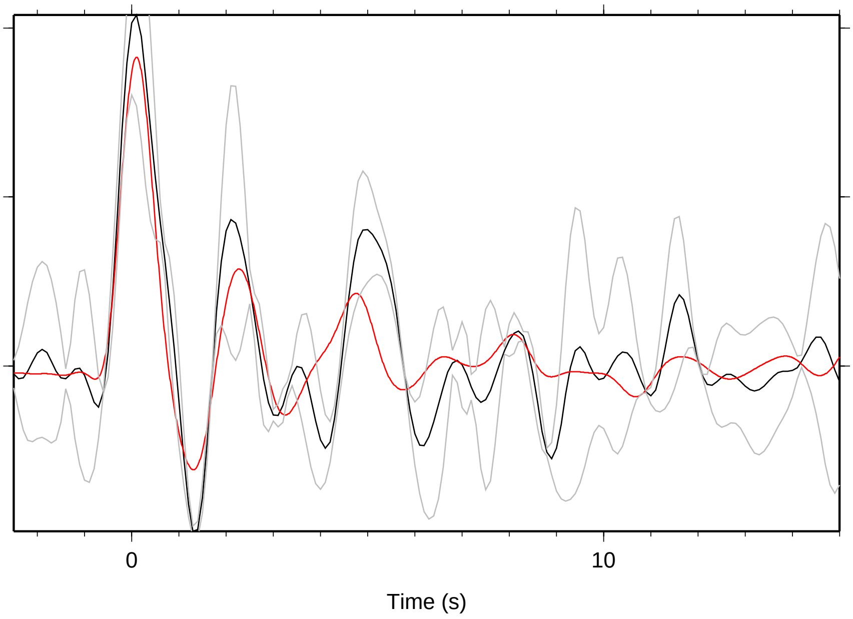

Supplementary Figure 30: Comparison of data RF (black) and model RF (red) for SKI (St. Kitts). The grey lines show the pointwise 2σ-jackknife uncertainties.

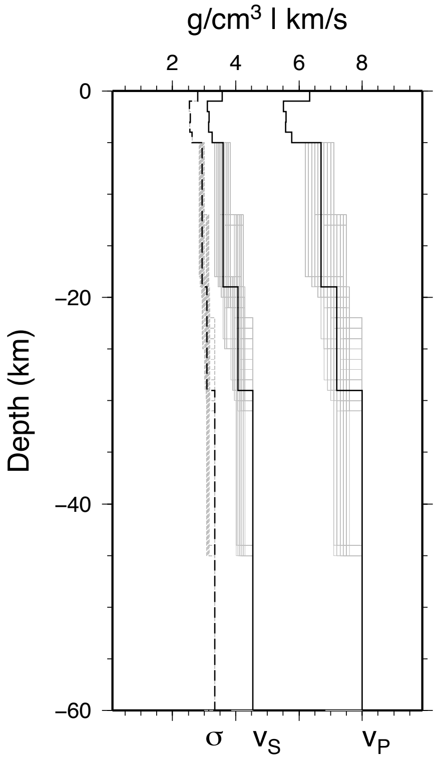

Supplementary Figure 31: 1-D modelling profile for SVB (St. Vincent). See Supplementary Figure 13 for further information.

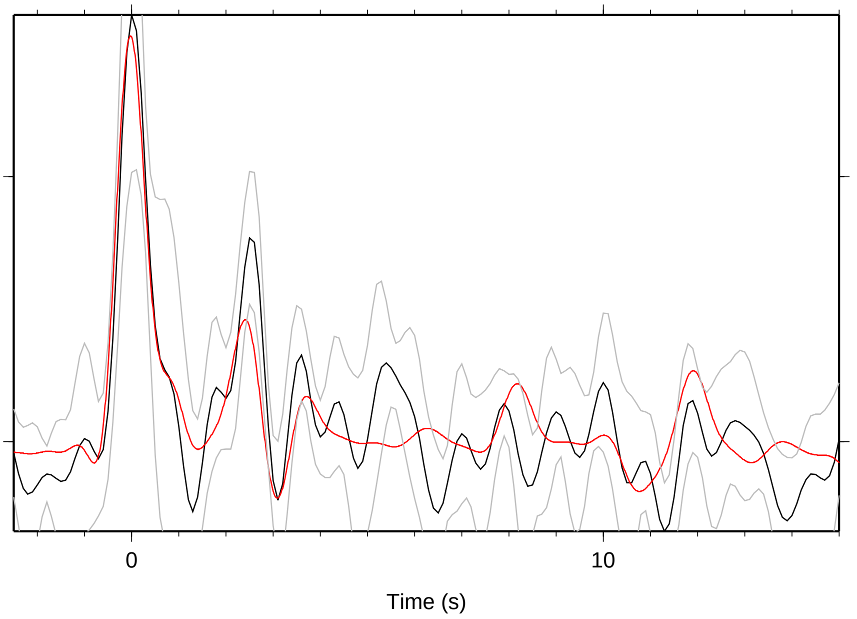

Supplementary Figure 32: Comparison of data RF (black) and model RF (red) for SVB (St. Vincent). The grey lines show the pointwise 2σ-jackknife uncertainties.

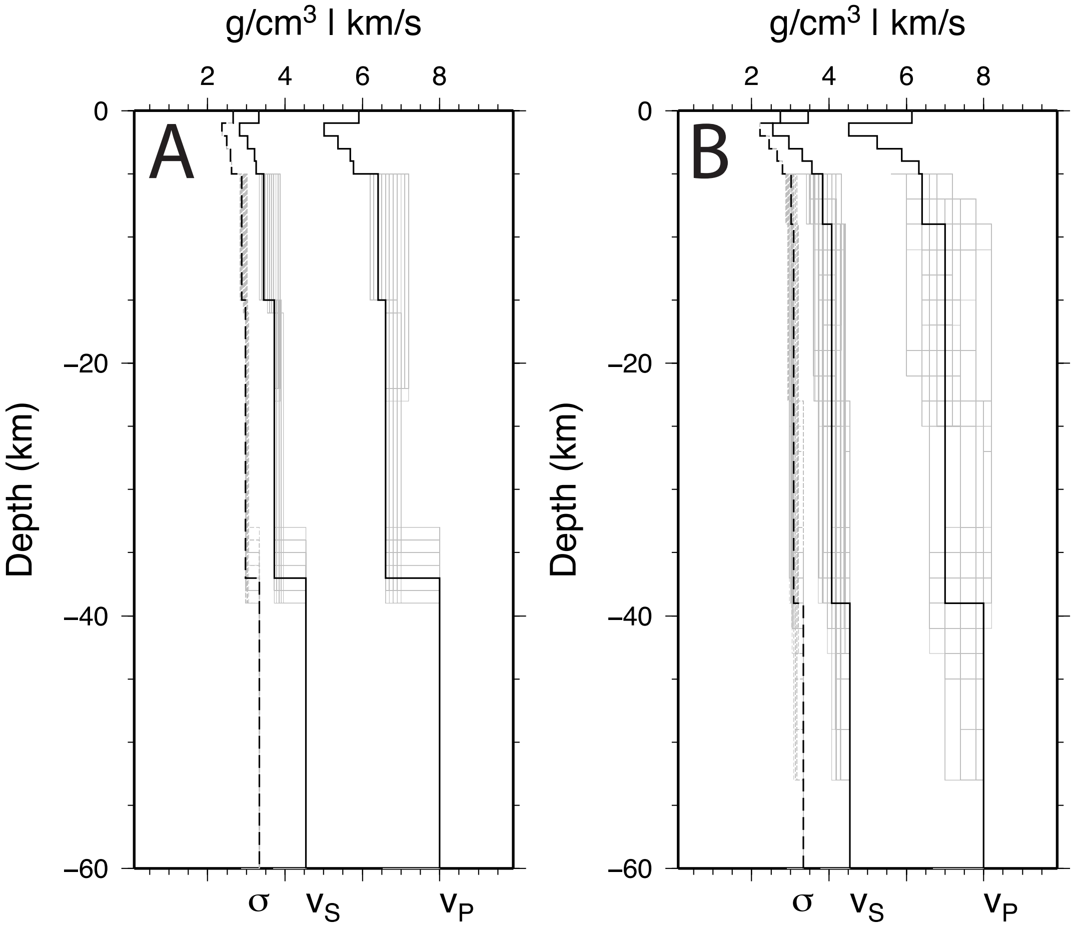

Supplementary Figure 33: Comparison of 1-D modelling profiles for station DHS with (A) and without (B) petrological constraints. Note that due to the much larger parameter space the parameter increments for the grid-search in (B) are increased by 100%.

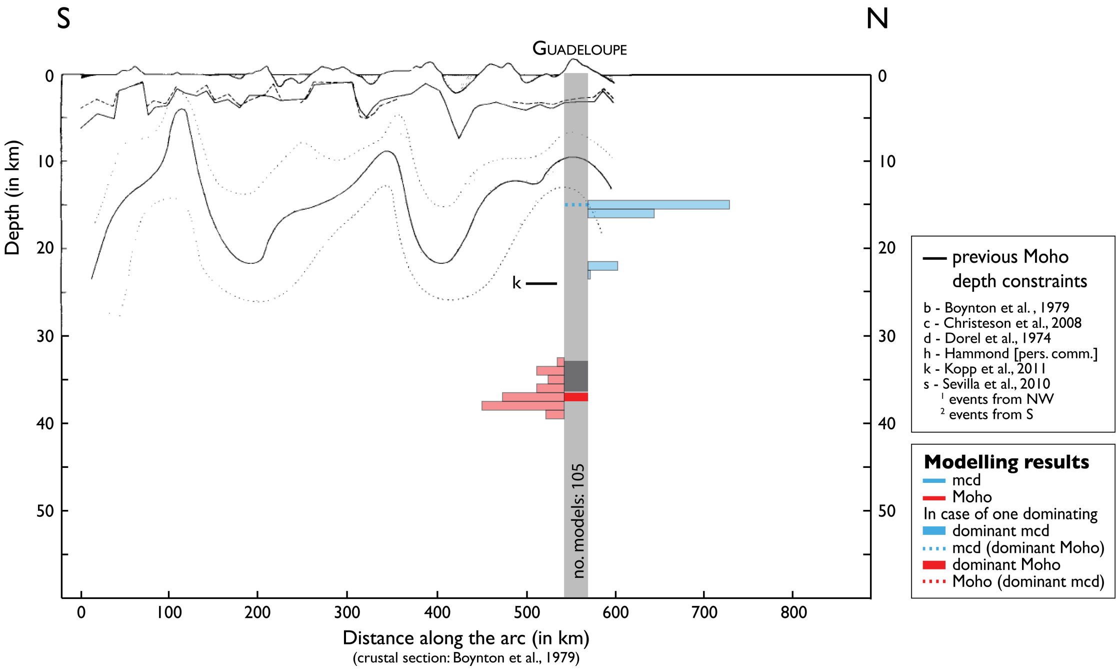

Supplementary Figure 34: Overview over all modelling solutions for DHS (Guadeloupe). The blue and red bars represent MCD and Moho depth results, the bar lengths are proportional to the occurrence frequency of that solution. The best modelling solution is depicted by the blue and red bars on top of the grey vertical bar. The darker section of the grey vertical bar shows the H-κ solution and its uncertainty. The number of models with a goodness-of-fit above 95% to the best model is shown at the bottom.

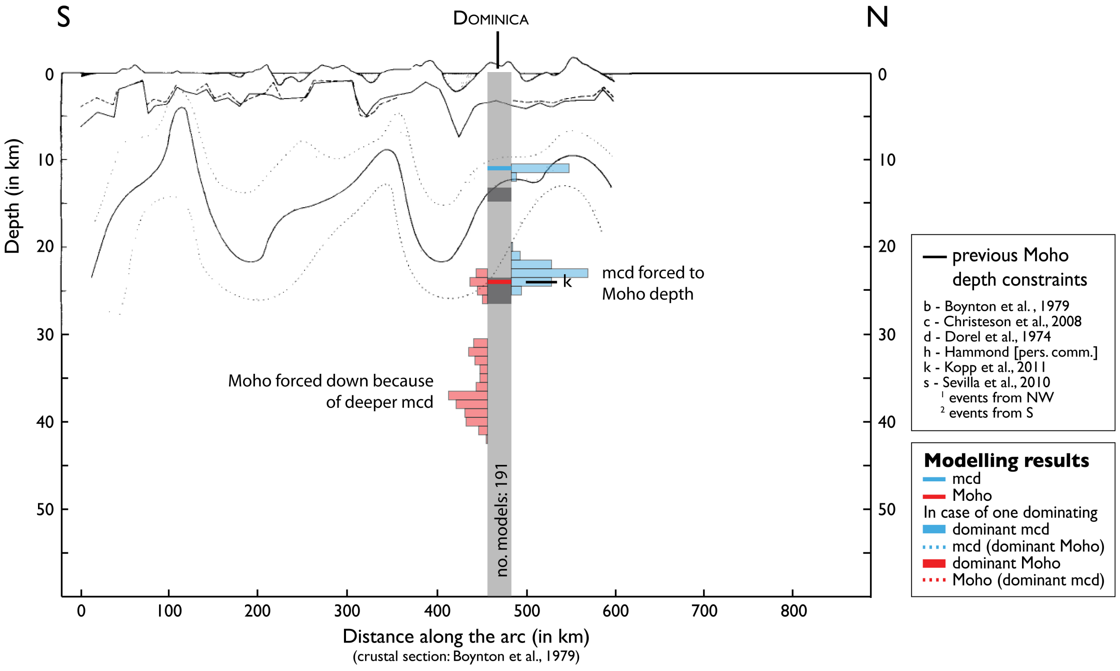

Supplementary Figure 35: Overview over all modelling solutions for DLPL (Dominica), all events except from NW direction. See Supplementary Figure **??** for further information.

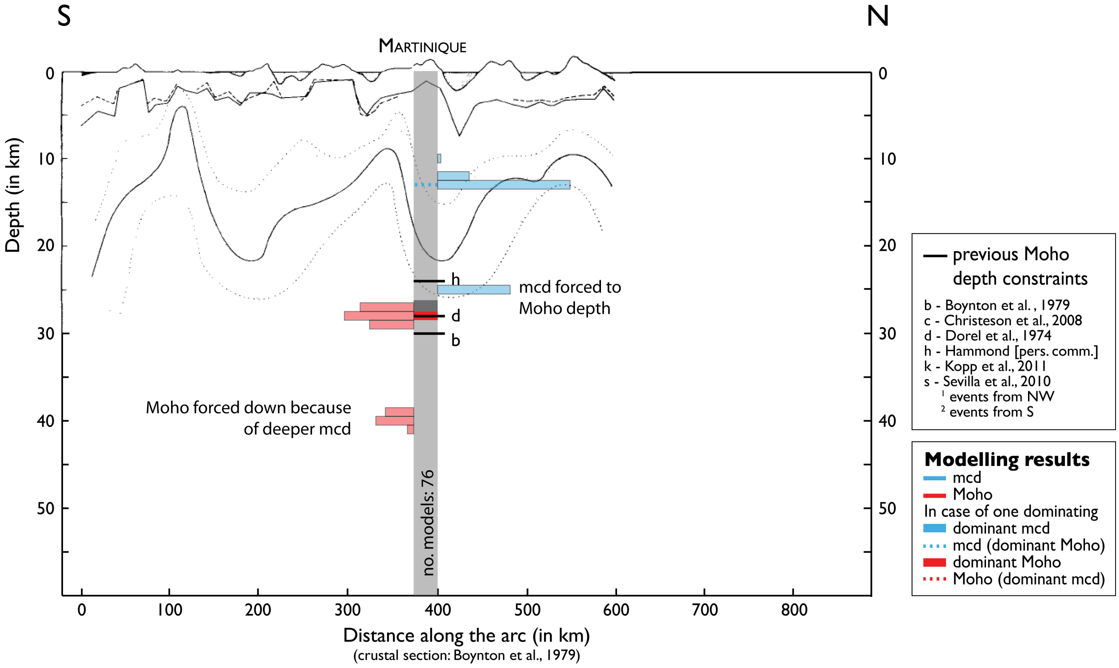

Supplementary Figure 36: Overview over all modelling solutions for FDF (Martinique). See Supplementary Figure **??** for further information.

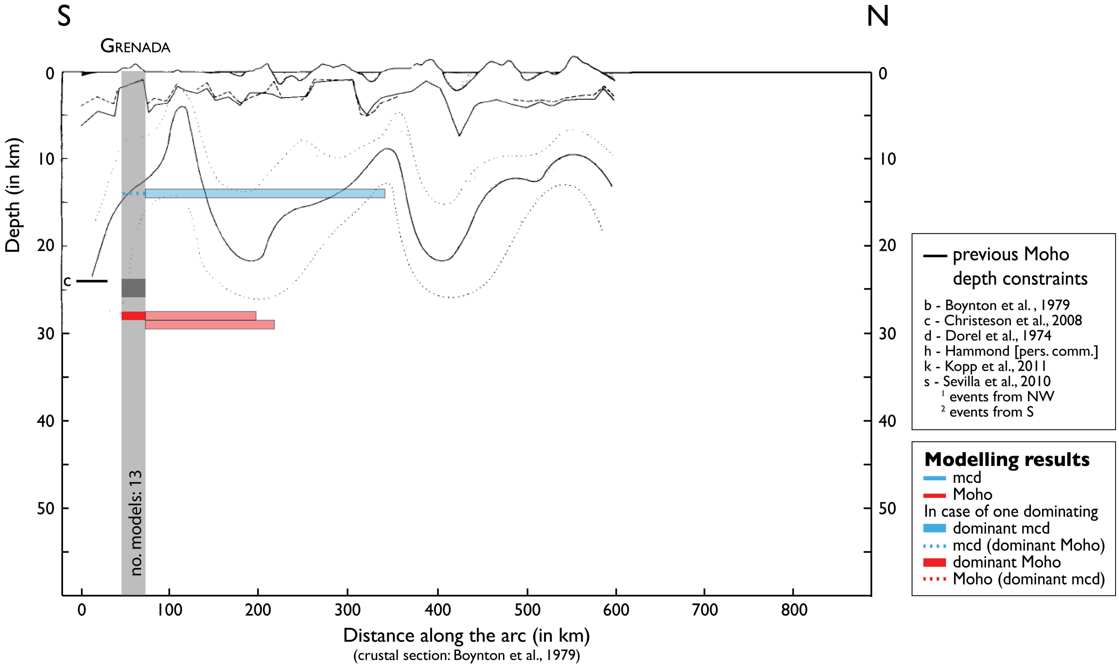

Supplementary Figure 37: Overview over all modelling solutions for GRGR (Grenada). See Supplementary Figure **??** for further information.

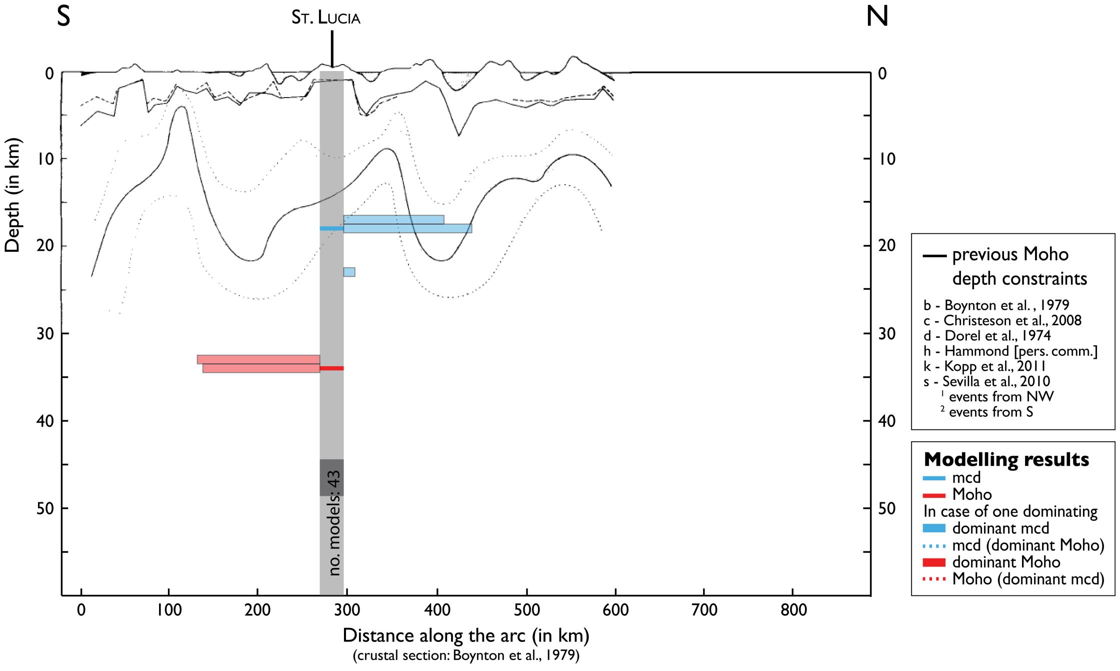

Supplementary Figure 38: Overview over all modelling solutions for MCLT (St. Lucia). See Supplementary Figure **??** for further information.

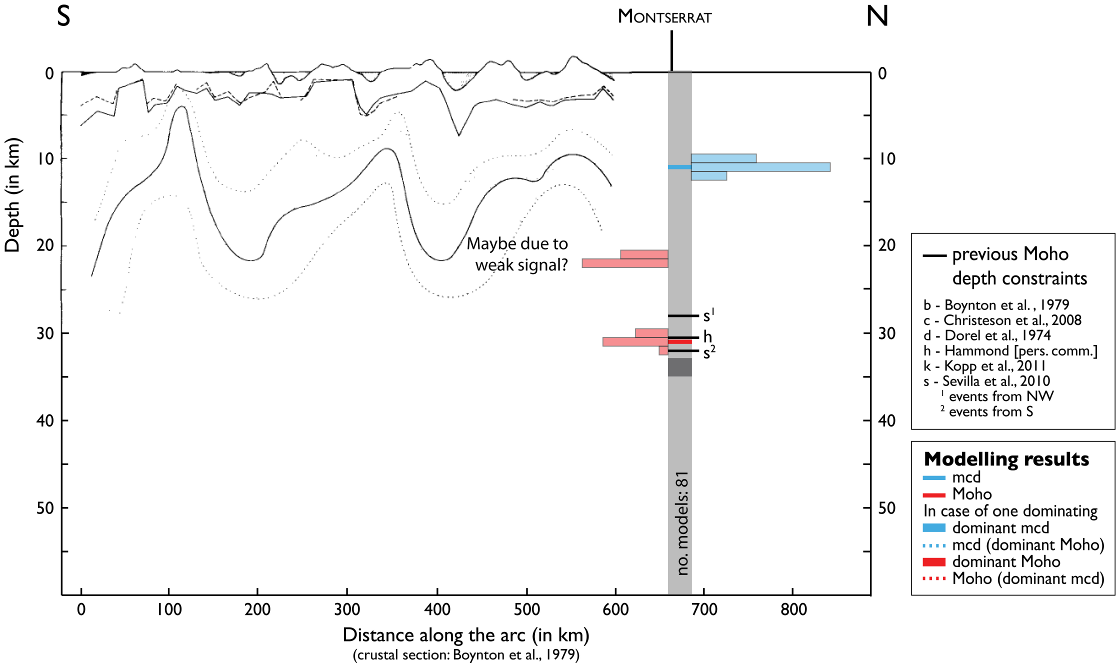

Supplementary Figure 39: Overview over all modelling solutions for all Montserrat stations combined. See Supplementary Figure **??** for further information.

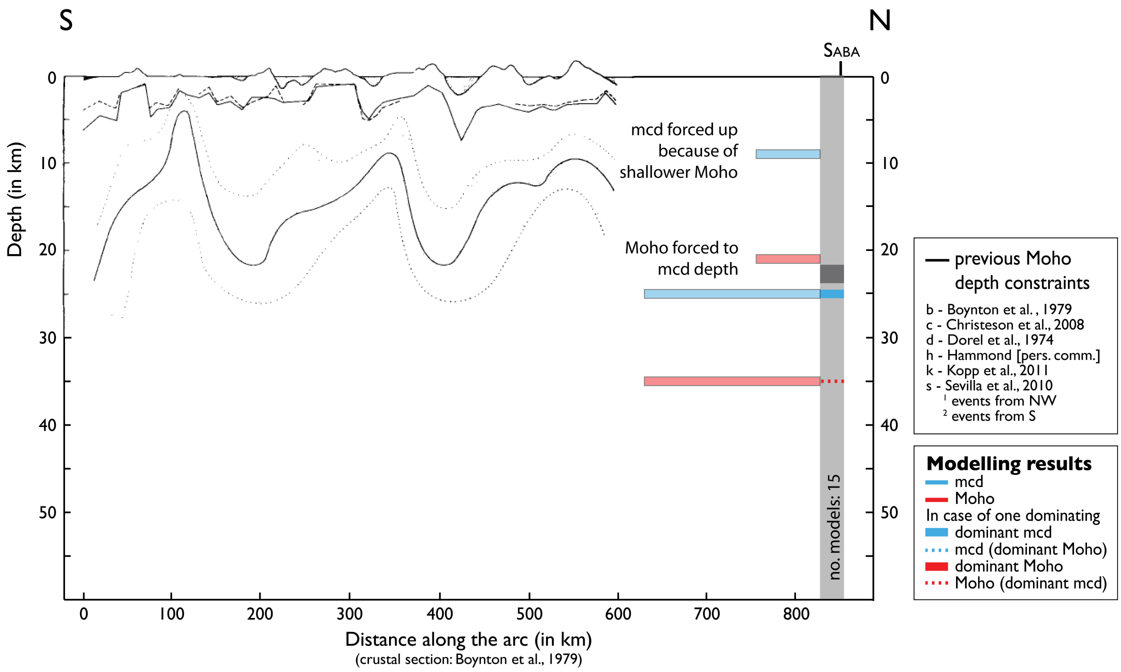

Supplementary Figure 40: Overview over all modelling solutions for SABA (Saba). See Supplementary Figure **??** for further information.

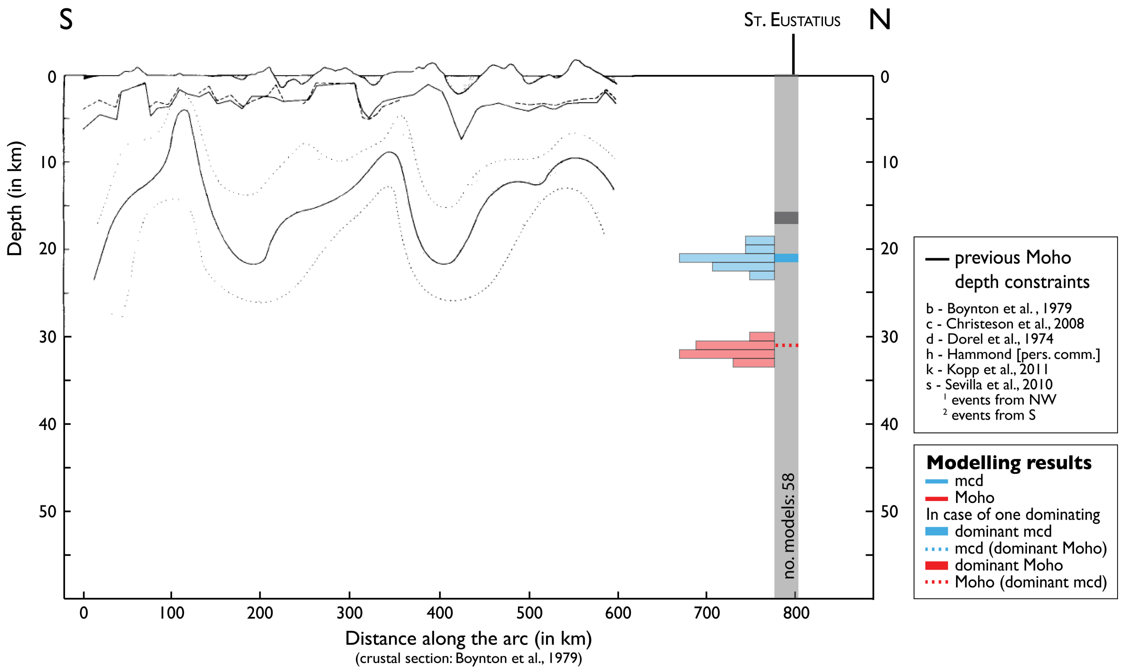

Supplementary Figure 41: Overview over all modelling solutions for SEUS (St. Eustatius). See Supplementary Figure **??** for further information.

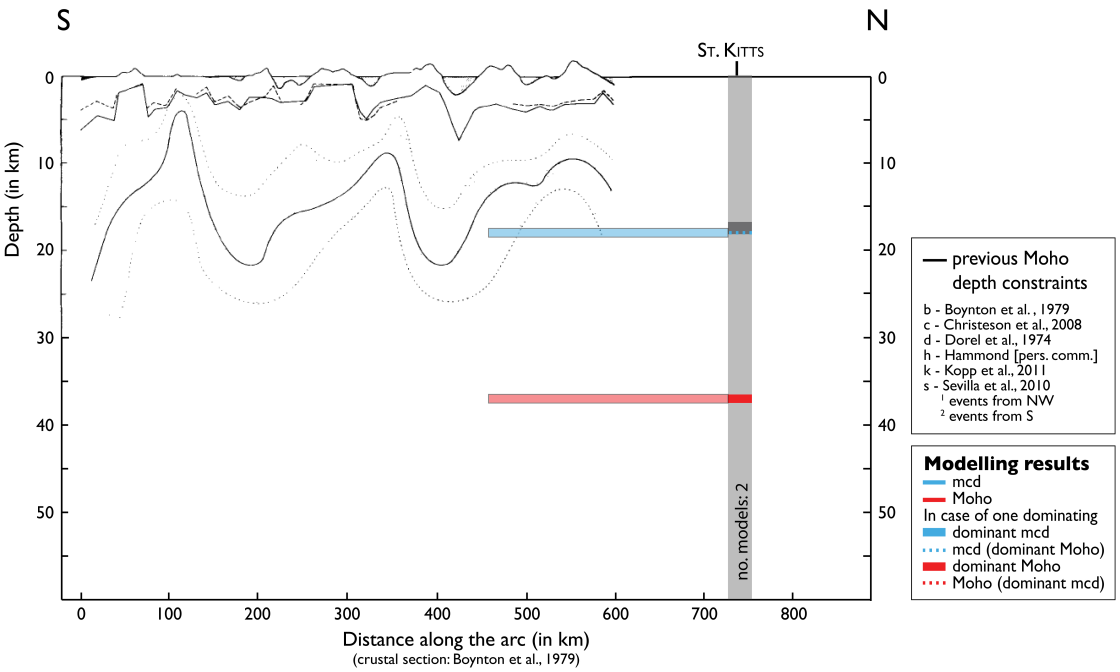

Supplementary Figure 42: Overview over all modelling solutions for SKI (St. Kitts). See Supplementary Figure **??** for further information.

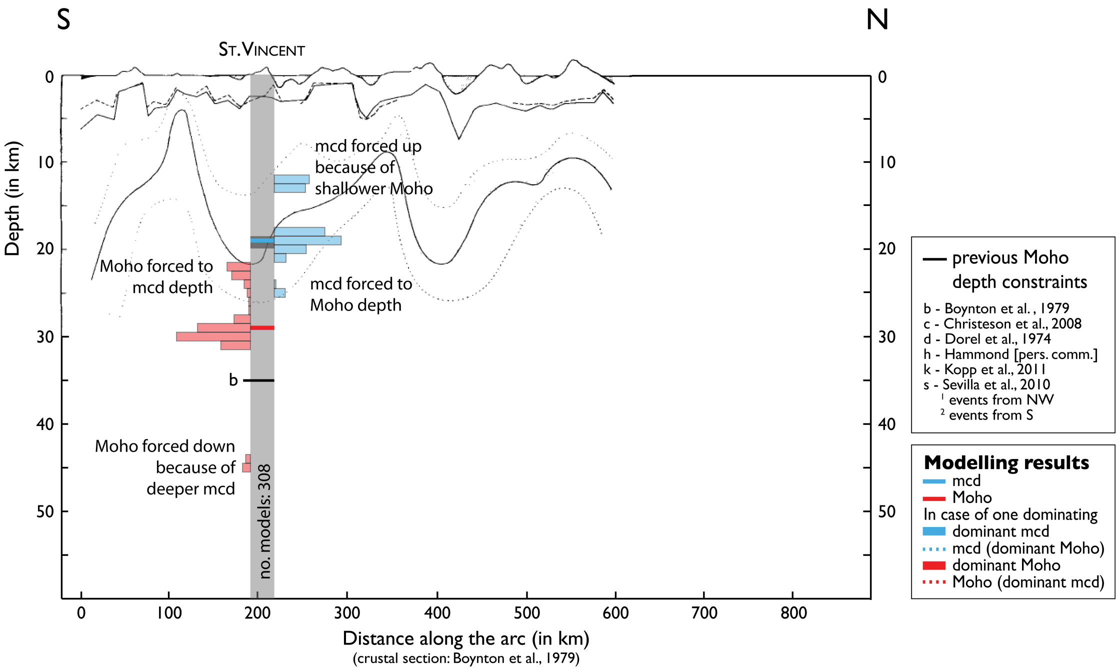

Supplementary Figure 43: Overview over all modelling solutions for SVB (St. Vincent). See Supplementary Figure **??** for further information.
