# Supplementary material for: Probing layered arc crust in the Lesser Antilles using receiver functions
Source: R Soc Open Sci. 2018 Nov 14;5(11):180764. doi: 10.1098/rsos.180764 (PMC6281922; doi:10.1098/rsos.180764)
Supplement: Infographic [file rsos180764supp3.pdf]

Improving The Resolution Of Layered Crust In Lesser Antilles Arc (LAA)

Data from 26 seismic stations on 10 islands was used to study crustal discontinuities along LAA

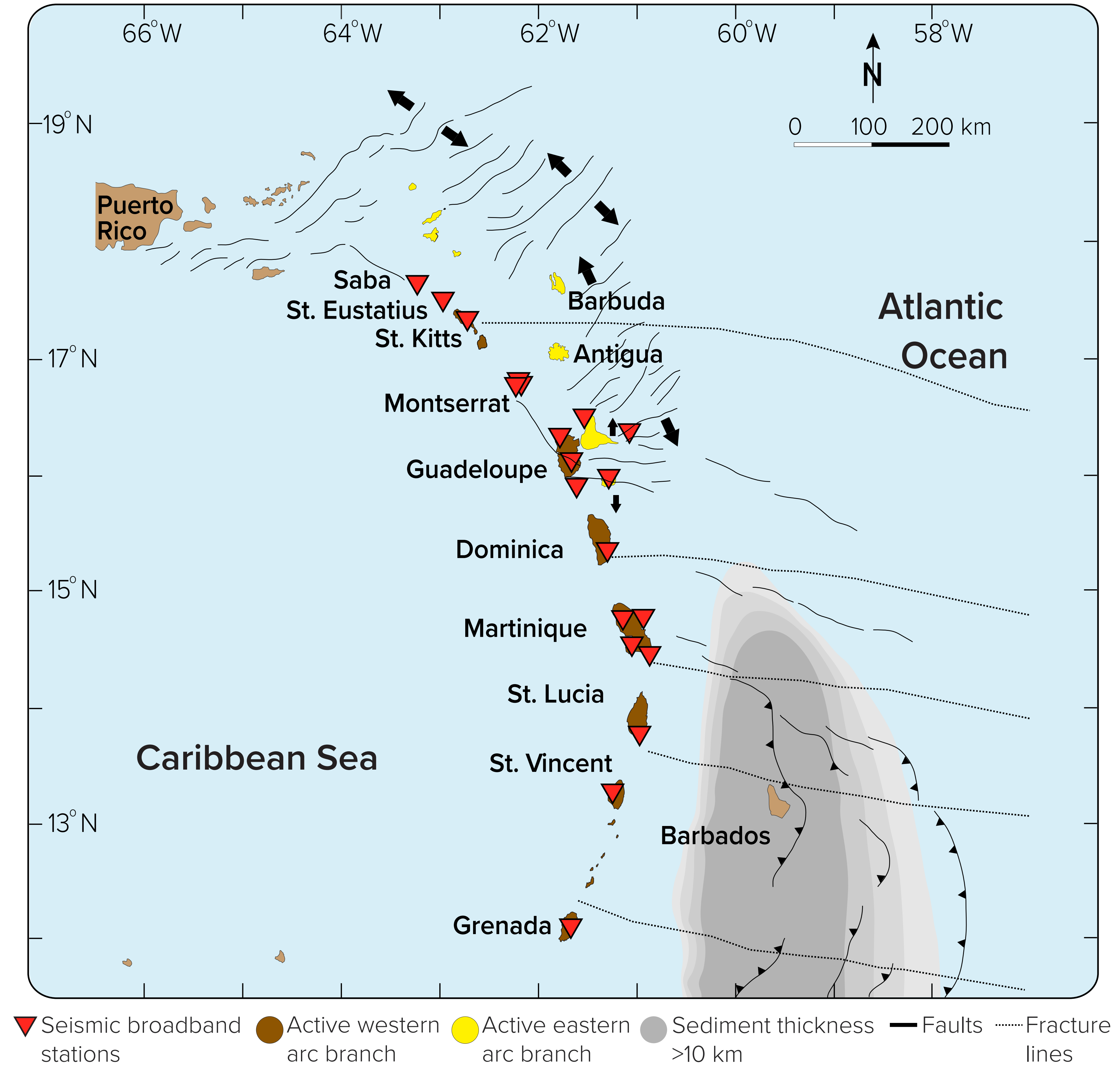

Inversion modelling approach integrating seismic and petrological results was used to circumvent misinterpretations of the former

Modelling approach used showed comparable depth estimates of the crustal discontinuities to those reported by previous studies

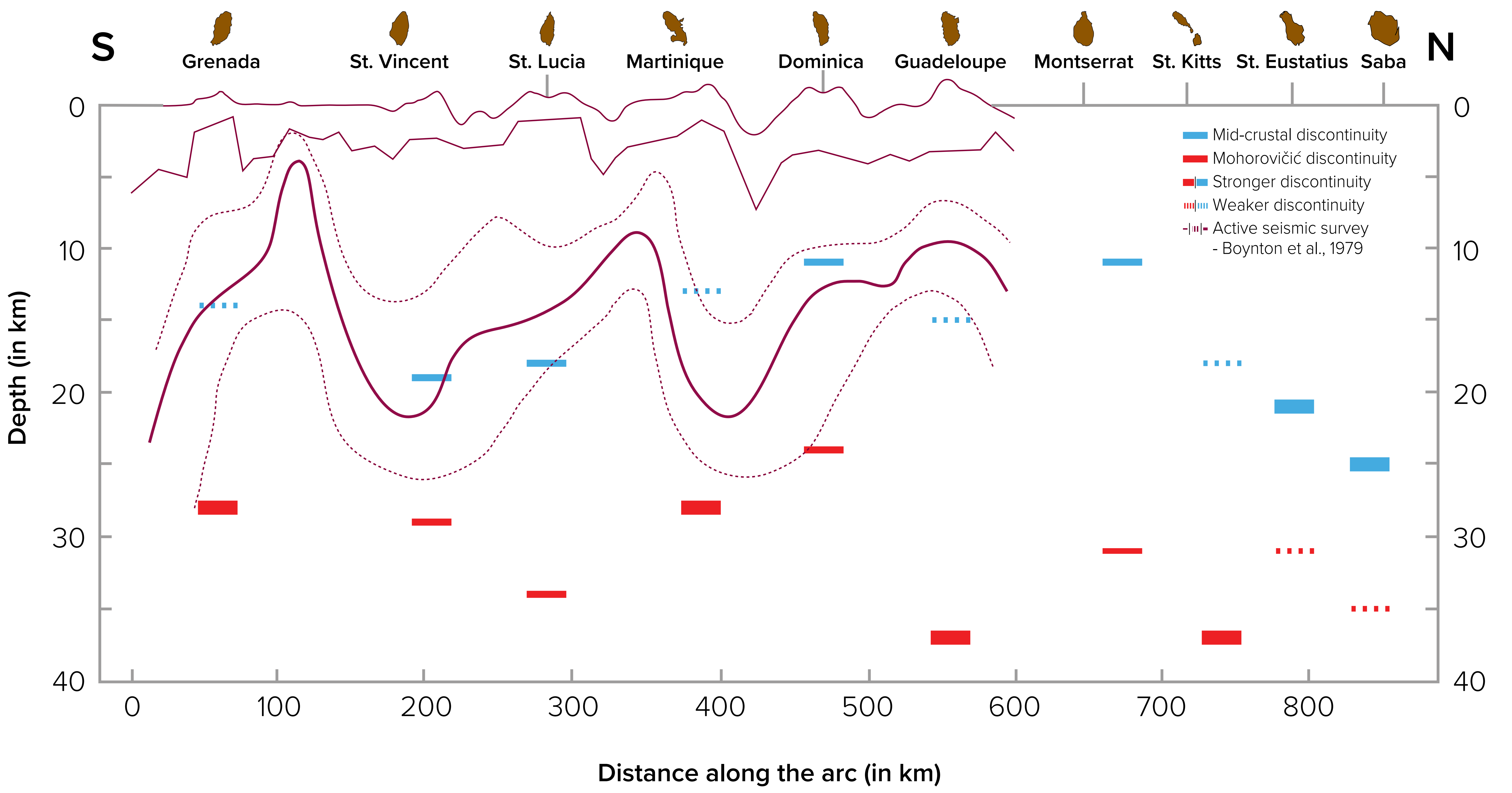

Results also indicated differences in magmatic water content and differentiation history of each island

Integration of seismological and petrological results enabled detailed study of crustal structure of all major islands in LAA

THE ROYAL SOCIETY PUBLISHING
ROYAL SOCIETY OPEN SCIENCE

Article: *Probing Layered Arc Crust in the Lesser Antilles using Receiver Functions* by Schlaphorst *et al.* 2018
This infographic was created as a collaborative effort between the journal article authors and Editage.
